# Rice OsCASP1 orchestrates Casparian strip formation and suberin deposition in small lateral roots to maintain nutrient homeostasis

**DOI:** 10.3389/fpls.2022.1007300

**Published:** 2022-12-19

**Authors:** Xianfeng Yang, Huifang Xie, Qunqing Weng, Kangjing Liang, Xiujuan Zheng, Yuchun Guo, Xinli Sun

**Affiliations:** Key Laboratory of Ministry of Education for Genetics, Breeding and Multiple Utilization of Crops, College of Agriculture, Fujian Agriculture and Forestry University, Fuzhou, China

**Keywords:** CASP, exodermis, endodermis, sclerenchyma, leaf senescence, ion, salt stress

## Abstract

*Arabidopsis* Casparian strip membrane domain proteins (CASPs) form a transmembrane scaffold to recruit lignin biosynthetic enzymes for Casparian strip (CS) formation. Rice is a semi-aquatic plant with a more complex root structure than *Arabidopsis* to adapt its growing conditions, where the different deposition of lignin and suberin is crucial for adaptive responses. Here, we observed the structure of rice primary and small lateral roots (SLRs), particularly the deposition patterns of lignin and suberin in wild type and *Oscasp1 mutants*. We found that the appearance time and structure of CS in the roots of rice are different from those of *Arabidopsis* and observed suberin deposition in the sclerenchyma in wild type roots. Rice CASP1 is highly similar to AtCASPs, but its expression is concentrated in SLR tips and can be induced by salt stress especially in the steles. The loss of OsCASP1 function alters the expression of the genes involved in suberin biosynthesis and the deposition of suberin in the endodermis and sclerenchyma and leads to delayed CS formation and uneven lignin deposition in SLRs. These different depositions may alter nutrient uptake, resulting in ion imbalance in plant, withered leaves, fewer tillers, and reduced tolerance to salt stress. Our findings suggest that OsCASP1 could play an important role in nutrient homeostasis and adaptation to the growth environment.

## Introduction

Plant roots acquire nutrients from soil and transport them across all external cell layers into the central vasculature and then upward to the aerial parts. Water and nutrients move radially into the stele through a combination of three pathways. The first is the apoplastic pathway, where solutes diffuse in free spaces and cell walls of the epidermis and cortex, which can be completely blocked by Casparian strips (CS) (Barberon and Geldner, 2014; Doblas et al., 2017a; Barberon, 2017). The second is the symplastic pathway involving cell-to-cell transport *via* plasmodesmata, and the third is the coupled transcellular pathway, where polarized influx and efflux carriers transport solutes in a vectorial fashion (Barberon and Geldner, 2014; Doblas et al., 2017a; Barberon, 2017). The solutes obstructed by CS are transported into endodermal cells by relevant influx carriers and then move into the stele *via* efflux carriers and/or plasmodesmata. Suberin lamellae do not block apoplastic transport but rather limit transcellular transport of nutrients (Doblas et al., 2017a), which coat the entire endodermal cell surface between the plasma membrane and secondary cell wall and isolate the solute from carriers (Robbins II et al., 2014).

CS formation is initiated at Casparian strip domain proteins (CASPs) at the Casparian strip membrane domain (CSD) in *Arabidopsis*. LORD OF THE RINGS 1 (LOTR1), a putative extracellular protease, is a crucial regulator in CSD positioning (Kolbeck et al., 2022). AtCASPs form a scaffold or a stable matrix at CSD for the subsequent lignification machinery (Roppolo et al., 2011; Lee et al., 2013). The transcription factor MYB36 controls the expression of the main genes involved in CS establishment, and AtCASPs recruit secreted proteins to the CSD, such as ENHANCED SUBERIN 1 (ESB1), Peroxidase 64 (PER64), and RESPIRATORY BURST OXIDASE HOMOLOG F (RbohF), to form localized lignin depositions (Doblas, Geldner and Barberon, 2017). SCHENGEN3/GASSHO1 (SGN3/GSO1) and SGN1 modulate the fusion of the CS into a continuous ring, which is constantly checked by two small peptides (Casparian strip integrity factor 1/2, CIF1/2) produced in the vasculature that diffuse into the apoplastic space in order to test endodermal barrier integrity (Nakayama et al., 2017; Wang P. et al., 2019). A similar mechanism of CS formation was uncovered in tomato (Li et al., 2018). Three OsMYB36 members redundantly regulate multiple genes implicated in CS formation at the root endodermis (Wang Z. et al., 2022). Maize Salt-Tolerant Locus 1 (*ZmSTL1*) encodes a dirigent protein localized at the CSD, and its mutation impairs lignin deposition at endodermal CS domain which leads to a defective CS barrier (Wang Y. et al., 2022).

Endodermal suberin plays crucial roles in plant nutrition by forming barriers for the free diffusion of water and nutrients. Deposition of suberin requires biosynthesis of aliphatic, phenolic and glycerol monomers, and transportation to the cell wall to form an insoluble macromolecular assembly (Woolfson et al., 2022). Those *Arabidopsis* genes involved in and regulating suberin biosynthesis were summarized in **Table S1**. Arabidopsis 3-ketoacyl-CoA synthase 2 (AtKCS2) and AtKCS20 is responsible for the production of long-chain fatty acids (Lee et al., 2009), CYTOCHROME P450, FAMILY 86, SUBFAMILY A1 (CYP86A1) and CYP86B1 are required for ω-hydroxy acid biosynthesis (Hofer et al., 2008; Compagnon et al., 2009), Fatty acyl-coenzyme A reductase 1 (FAR1), FAR4, and FAR5 are involved in the production of different chain-length primary fatty alcohols (Domergue et al., 2010), GLYCEROL-3-PHOSPHATE ACYLTRANSFERASE 5 (GPAT5) is involved in the synthesis of monoacylglycerol esters (Beisson et al., 2007), and ALIPHATIC SUBERIN FERULOYL TRANSFERASE (ASFT), ATP binding cassette transporter subfamily G2 (ABCG2), AtABCG6, and AtABCG20 transfer from feruloyl-CoA to ω-hydroxy acids and fatty alcohols (Molina et al., 2009; Yadav et al., 2014). Several Myb transcription factors, including *Myb41, Myb53, Myb92*, *Myb93*, and *Myb39*, have been identified to regulate suberin deposition by modulating the expression of suberin biosynthetic genes (Kosma et al., 2014; Wang C. et al., 2020; Shukla et al., 2021). Several homologous genes in potato (*Solanum tuberosum*) show similar functions to that in *Arabidopsis* (Woolfson et al., 2022). The rice transporter REDUCED CULM NUMBER1 (RCN1/OsABCG5) is involved in the suberization of the hypodermis/exodermis of rice roots (Shiono et al., 2014). In addition, suberization of endodermal cells responds to a wide range of nutrient stresses, is induced by ABA, and is depressed by ethylene (Barberon et al., 2016).

CS formation and suberin deposition are interrelated. The CS defective mutants, excluding *sgn3*, show ectopic suberin deposition in *Arabidopsis* and usually alter the ion permeability and sensitivity to salt and drought stress (Doblas et al, 2017a; Barberon, 2017). These ectopic endodermal lignification and suberization could act as compensation and are triggered through the endodermal integrity control system consisting of SGN3 and CIF1/2 (Doblas et al., 2017b; Fujita et al., 2020; Okuda et al., 2020). CIF1/2 and ABA treatment enhances the expression of *Myb41, Myb53, Myb92*, and *Myb93*, and ABA and SGN3/CIFs pathways can induce ectopic endodermal suberization by regulating the expression of these *Myb* genes (Wang C. et al., 2020).

There are 5 CASPs and 34 CASP-likes (CASPLs) in *Arabidopsis*, and CASPLs should have a conserved module for membrane subdomain formation and are expressed in a tissue-specific manner. At least twelve AtCASPLs are able to reach the plasma membrane under the control of the AtCASP1 promoter, and nine of these are clearly located at the CSD just like AtCASPs. These results address different cell wall-modifying machineries in different tissues (Roppolo et al., 2014). However, the positions in the cell of the other CASPLs are less known. *AtCASPL4C1* knock-out plants shows earlier flowering compared to wild type and overexpressing *CICASPL* (The ortholog of *AtCASPL4C1* in *Citrullus lanatus*) results in increased sensitivity to cold stress in *Arabidopsis* (Yang et al., 2015). There are 6 OsCASPs and 28 OsCASPLs in rice (**Figure S1**), and the function of OsCASP1 has recently been studied, which indicates that OsCASP1 is required for CS formation in endodermal cells (Wang Z. et al., 2019). OsCASP1 can form complexes with itself and OsCASP2, which leads to ectopic protein accumulation in rice cell under control of the 35S promoter (Wang Z. et al., 2020). The rice root system is more complex than that of *Arabidopsis*, and its radial structure includes the epidermis, exodermis, sclerenchyma, midcortex, endodermis, and stele from the outside inward (Rebouillat et al., 2009). There is no CS of the exodermis, sclerenchyma, and aerenchyma in *Arabidopsis* root (Rebouillat et al., 2009; Robbins II at al., 2014). These specified tissues could allow rice to adapt to its growth condition.

Here we report the discovery of a novel mutant, which exhibits withered leaf phenotype and fewer tillers comparing to the wild type. We discovered that the loss of OsCASP1 function results in the mutant phenotype using map-based cloning. OsCASP1 shows high sequence similarity to AtCASPs (**Figure S1**), is highly upregulated at small lateral root tips (SLRs), and is strongly expressed in roots, especially in the stele and sclerenchyma, after salt treatment. The loss of OsCASP1 function leads to delayed CS formation and ectopic suberin deposition in SLRs and alters ion balance in plants.

## Materials and methods

### Constructions for the complementation test and tissue-specific expression

Recombinant plasmids were constructed using the In-Fusion cloning kit (Takara). To generate *OsCASP1pro:OsCASP1*, the *OsCASP1* promoter (1128 bp upstream of the *OsCASP1* gene including the whole intergenic sequence between *Os04g0684200* and *OsCASP1*) and gene was amplified, and the PCR product was recombined into pCAMBIA-1300 digested with Hind III with the In-Fusion cloning kit. To generate *OsCASP1pro:OsCASP1-GUS*, the *OsCASP1* promoter and gene was amplified and recombined into pCXGUS-P digested with Xcm I, respectively. The details of these constructs are shown in **Figure S2**, and the primers used in this study were shown in **Table S2**.

To perform the complementation experiment, some seeds were first collected from a few F2 individuals, which exhibited a mutant phenotype and a Nipponbare-like morphology, from a cross between the *Oscasp1-3* mutant and Nipponbare; the construct of *OsCASP1pro:OsCASP1* was transformed into the calli from these seeds; and the transgenic plants with the construct were observed and examined using PCR. The construct of *OsCASP1pro:OsCASP1-GUS* was transformed into Zhonghua 11 (ZH11).

### Plant materials and growth conditions

In the study, three *Oscasp1* mutants were used. The first is *Oscasp1-3*, a natural mutant in a paddy field, which is derived from a high generation progeny of Jinhui2629 and TR-2 with an *indica* genetic background. The second is *Oscasp1-4*, a CRISPR/Cas9 mutant, which was generated by the Biogle Company. The CRISPR target sequence was localized to exon 1 of the *OsCASP1* gene. Sixteen mutant lines were obtained, but only one line (*Oscasp1-4*) survived. The third is *Oscasp1-1* with a Nipponbare background (Wang Z. et al., 2019), which was kindly provided by Dr. Jixing Xia (Guangxi University, Nanning, China).

Rice plants were grown under two different conditions: soil and hydroponics. For soil experiments, the F_2_ and F_3_ populations of the cross of the *Oscasp1-3* mutant and Nipponbare were grown in the experimental paddy field in Putian, Fujian province. For hydroponic experiments, rice seeds were soaked in a dark incubator (28°C) for 48h. After germination, seeds were grown in 1×Kimura B nutrient (**Table S2**) under a photoperiod of 28°C/14 h of light and 28°C/10 h of darkness. The seedlings were grown in a growth cabinet in the nutrient solution to test the sensitivity of the plants to nutrient deficiency.

### DNA and protein sequence analysis

The new molecular markers were designed according to the Nipponbare sequence, and the CASP and CASP-like proteins in rice were obtained by searching the RAP-DB database with the BlastP programmes. The Molecular Evolutionary Genetic Analysis programme (MEGA X) was used to generate phylogenetic trees using the maximum likelihood method and JTT matrix-based model (Kumar et al., 2018).

### Histochemical staining

Roots of 5~12-d-old seedling were used for the study, and freehand cross-sections were cut at different regions. To observe the Casparian strip, root cross-sections were stained with 0.1% (w/v) berberine chloride (Sangon) and 0.5% (w/v) aniline blue (Sangon) as described by Brundrett et al. (Brundrett et al., 1988). Casparian strips were visualized as bright white/yellow fluorescence (UV filter set) under Nikon (MODEL ECLIPSE Ni-U) microscope. To visualize lignin with cinnamyl aldehyde groups in the roots, cross-sections were stained for 30 min with 1% phloroglucinol (Sangon) in 20% (w/v) hydrochloric acid at room temperature and observed using a Nikon microscope (MODEL ECLIPSE Ni-U). Lignin appears orange/red under white light (Pradhan Mitra and Loque, 2014; Shiono et al., 2014). Suberin deposition in the root was visualized with Fluorol Yellow 088 (ACMEC) as described by Barberon et al. (Barberon et al., 2016). Lignin in SLR was visualized with Basic Fuchsin (Sangon) or Auramine O (Sangon) in ClearSee solution as described by Urache et al. (Ursache et al., 2018). The excitation wavelength of FY088 and Auramine O is 488 nm, and the emission fluorescence was detected at 516-593 nm. The excitation wavelength of Basic Fuchsin is 561 nm, and the emission fluorescence was detected at 600-650 nm. Suberin or lignin deposition in roots was observed with the Leica TCS SP8 laser scanning confocal microscope.

### Electron microscopy

The endodermis was visualized using root sections collected 10 mm and 20 mm from the root tips in 17-d-old seedlings. Transmission electron microscopy (TEM) was conducted using a previously described protocol (Hulskamp et al., 2010). Samples were observed using the Hitachi HT-7800 transmission electron microscope operating at 20-120 kV.

### Permeability test

For the propidium iodide (488 nm excitation, 500-550 nm emission) (Sangon) penetration assay, the roots of 5~7-d-old seedlings were incubated in a fresh solution of 10 μg/ml, 50μg/ml, or 100μg/ml propidium iodide for the indicated time in the dark, and then rinsed twice in water. Ater staining, freehand sections or small lateral roots were then observed with the Leica TCS SP8 confocal laser-scanning microscope (Naseer et al., 2012). To quantify PI penetration, SLRs with similar diameter were selected in the middle region of the primary roots of 12-d-old seedling for observation.

### Elemental analysis

The leaves in the tillering period were collected and used for elemental analysis, among which the *Oscasp1-3* mutant showed a few cell death dots. The tissues were completely dried in an oven, and the samples were treated as described by Hosmani et al. (2013) (Hosmani et al., 2013; Wang Z. et al., 2019). Elemental analysis was performed on a CIC-260 ion chromatograph (K, Na, Mg, and Ca) and ICP-MS (Cd, As, Mo, Fe, and Mn), and nine elements were monitored.

## Results

### Identification of the mutant with withered leaves and few tillers

**Figure 1:**
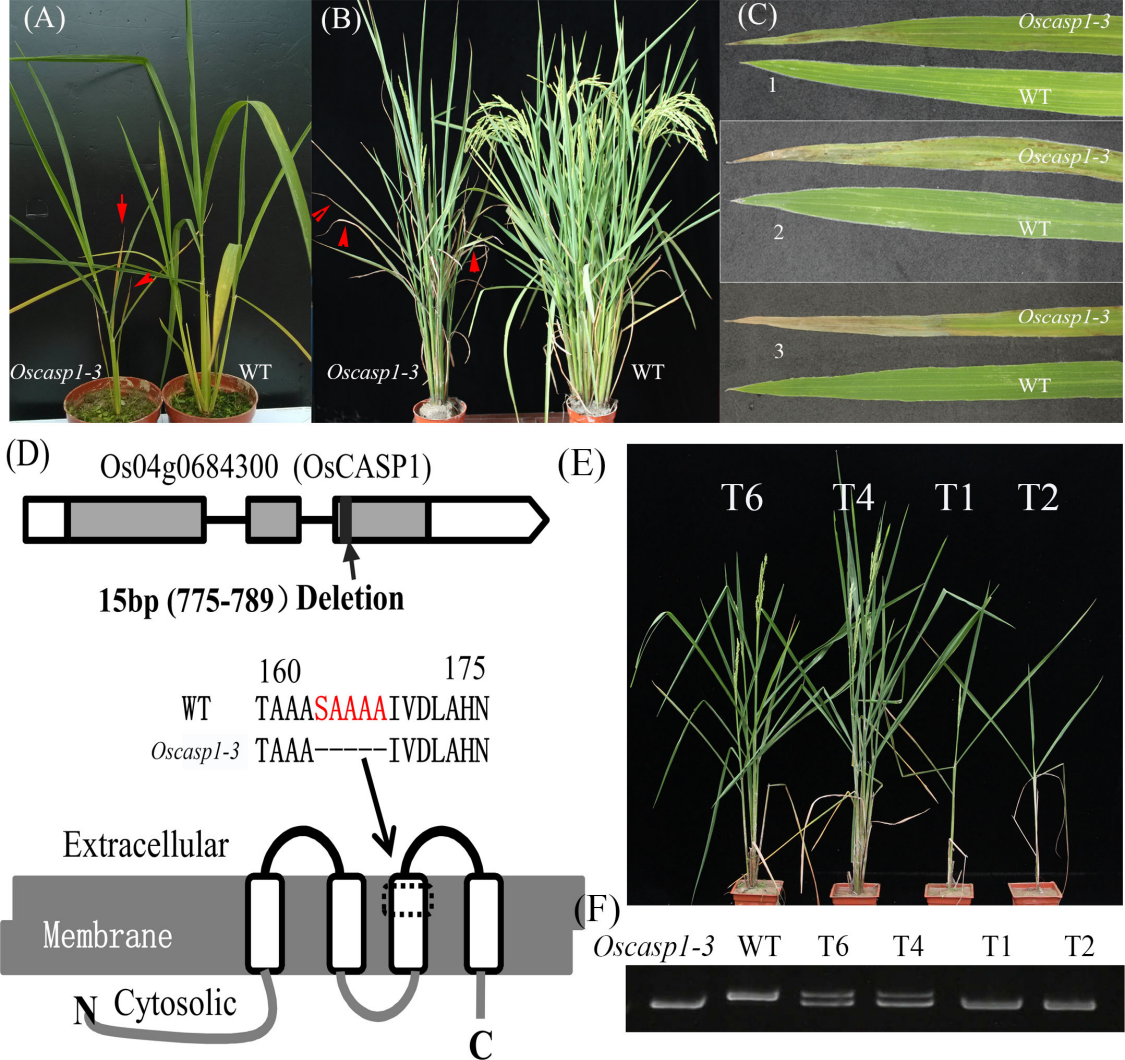
The phenotypes of the wild type and *Oscasp1-3* mutant and characterization of *Oscasp1-3*. **(A)** Seedlings of wild type and *Oscasp1-3* mutant plants. **(B)** The phenotypes of wild type and *Oscasp1-3* mutant plants at the heading stage. Red arrows indicate leaves with necrosis. **(C)** Phenotype of the leaves. 1, 2, and 3 indicate the relative positions from top to bottom. **(D)** The structure of OsCASP1 and deletion region in the *Oscasp1-3* mutant. **(E)** Phenotypic comparison of transgenic plants with the *OsCASP1* gene (T6 and T4) and segregant individuals without the *OsCASP1* gene (T1 and T2). **(F)** The PCR result indicated transgenic plants with or without the *OsCASP1* gene.

The mutant was discovered in a paddy field, came from a high generation progeny of Jinhui2629 and TR-2, and showed withered leaf phenotype and few tillers. The mutant and wild type (WT) used in the study showed stable inheritance. The mutant phenotype appears at the beginning of the tillering stage. Cell death usually begins at the blade tip or upper part of the leaf and then extends to the entire leaf as it grows. The mutant exhibits fewer tillers and shorter internodes at the adult stage relative to the WT (**Figures 1A–C** and **Figures S3A–D**).

To reveal the genetic basis of the mutant, we performed a cross between the mutant and Nipponbare and investigated 426 individual plants from the F_2_ population. Genetic analysis showed that a single recessive gene controls the mutant phenotype. We mapped the locus of interest on chromosome 4 and delimited it to a region of approximately 265-kb between the insertion-deletion (Indel) molecular markers ID4-3 and ID4-10. To further fine-map the mutant gene, we selected several F_2_ individual plants with a heterozygous genotype in the candidate region to establish the F_3_ population. The locus of interest was narrowed down to an 11.3-kb region between ID4-371 and ID4-3-8 using Indel markers, which contained two genes - *Os04g0684200* and *Os04g0684300* (**Figure S3E**). We sequenced the two genes and found a 15-bp deletion in the coding region of the *Os04g0684300* gene and no mutation in the *Os04g0684200* gene in the mutant (**Figure 1D**). *Os04g0684300* encodes a Casparian strip membrane domain protein1 (CASP1), and the mutation of *Os04g0684300* could cause withered leaves and few tillers.

To further validate CASP1 function, we performed genetic complementation by introducing the *OsCASP1* gene with its promoter into the calli from F_3_ seeds, which arose from a few individuals that exhibited mutant and Nipponbare-like phenotypes from the F_2_ population. We found that all transgenic lines carrying the *OsCASP1* gene have a restored WT phenotype (**Figures 1E, F**), which confirmed that OsCASP1 regulates the leaf senescence and tiller development. We therefore named the mutant *Oscasp1-3*.

We also generated transgenic plants with knockout *OsCASP1* gene using CRISPR/Cas9 technology. Most transgenic plants with the homozygous genotype exhibited obvious withered leaf phenotype, sterility, or eventual death (**Figure S4**), with only one line (*Oscasp1-4*) surviving at the end (**Figure S4C**). Some of transgenic lines with the heterozygous genotype also exhibited mutant phenotype, while further analysis showed that both alleles at the *OsCASP1* locus contain mutant sites (**Figure S4A**). These results suggest that the mutation of *OsCASP1* gene leads to the mutant phenotype.

### Pattern and induction of *OsCASP1* gene expression

**Figure 2:**
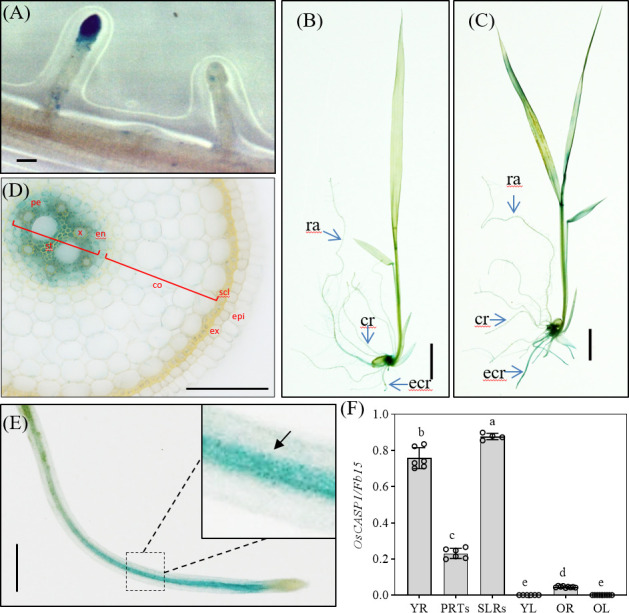
Localization of *OsCASP1pro:OsCASP1-GUS* expression. **(A)** GUS activity in SLR tip. **(B)** GUS staining of whole seedling plant grown in 1×Kimura B solution. **(C)** GUS staining of the plant treated with 100 mM NaCl for 3 hours. **(D)** Cross-section of a crown root (10 mm distal from the root tip) derived from a 10-d-old seedling treated with NaCl. **(E)** GUS activity in SLR treated with NaCl. **(F)** Expression levels of *OsCASP1* in different tissues. YR, young root. PRTs, primary root tips. SLRs, small lateral roots. YL, young leaves. OR, old roots. OL, old leaves. Different letters denote significant differences in one-way ANOVA test (Tukey test p < 0.05) among different lines. *Fb15* (Fiber protein 15) (Os02g0175800) was used as reference gene in the RT-qPCR analysis. Scale bars: **(A, D, E)** 100μm; **(B, C)** 1cm.

*Arabidopsis* CASPs were thought to control the formation of CS, and they are specially expressed at CS domain in roots. To further reveal the function of OsCASP1, we examined GUS activation in transgenic lines carrying *OsCASP1pro:OsCASP1-GUS* to reveal tissue-specific expression of *OsCASP1* and observed an intense blue color at the tips of SLRs, but not all tips of SLRs (**Figure 2A**). Salt stress strongly induced *OsCASP1* expression in roots and leaves (**Figures 2B, C**, and **S5**). We examined the cross and longitudinal sections of primary root after staining and found that GUS activity was mainly concentrated in the stele of roots (**Figures 2D** and **S5D–E**). We also uncovered *OsCASP1* gene expression in other root tissues treated with NaCl, especially sclerenchyma cells (**Figures 2** and **Figure S5**). The *OsCASP1* gene was highly upregulated in SLR tips and steles after NaCl treatment (**Figures 2E** and **S5A**). We also accessed the expression levels using RT-qPCR in different tissues and uncovered that *OsCASP1* is highly expressed in SLRs and younger roots, moderately expressed in primary root tip, and weakly expressed in leaves (**Figure 2F**).

### Characterization and CS structure of the WT and *Oscasp1* mutant in primary roots

In *Arabidopsis*, the *casp1-1/casp3-1* mutant shows abnormal CS structure and delayed establishment of a functional apoplastic barrier (Hosmani et al., 2013). OsCASP1 exhibits high sequence similarity with AtCASP1~4 (**Figure S1**), therefore it seems reasonable that OsCASP1 can regulate the formation of CS in rice. We first observed the structures of the primary roots (embryonic crown roots and postembryonic crown roots) and large lateral roots of the *Oscasp1-3* mutant after staining with phloroglucinol and berberine-aniline blue, respectively. We did not find obvious differences in root structure between the *Oscasp1-3* mutant and WT, including CS structure by observing the cross-sections (**Figure S6**).

Basic Fuchsin was used to visualize CSs and the lignin-based cell wall impregnations of the endodermis in *Arabidopsis* (Vermeer et al., 2014, Doblas et al., 2017b, Kalmbach et al., 2017). However, we also used this method to visualize CS in cross-sections in the primary roots and discovered that it is difficult to locate CS in the endodermis based on the result of Basic Fuchsin staining, which displayed a different lignin deposition patterns compared with phloroglucinol and berberine-aniline blue staining (**Figures S6**, **S7**). The CS is at the radial walls of endodermal cells, but we discovered that lignin deposition was disrupted in many radial walls of endodermal cells and that there was an area of the radial walls that could not be stained with Basic Fuchsin (**Figure S7**). We suspected that some unknown components in rice cell walls could influence the staining. In addition, Basic Fuchsin strongly stained many outer peripheral wall and stele-facing wall of endodermal cells and cell wall of cortical cells (**Figure S7**). We observed cross-sections at 10mm, 20mm, and 30mm from root tip and found that the farther endodermal cells from root tip, the more cells with increased lignin deposition. Lignin deposition in the *Oscasp1-3* mutant was higher in endodermal cells than that in the WT at the same slice position (**Figure S7A**). The *Oscasp1-1* mutant (Nipponbare background) was used to study the function of *OsCASP1* gene in the previous report (Wang Z. et al., 2019). Thus, we also examined lignin deposition patterns in the roots of the *Oscasp1-1* and *Oscasp1-4* mutants after staining with Basic Fuchsin. However, we did not observe the difference between Nipponbare and *Oscasp1-1*, and ZH11 and *Oscasp1-4* (**Figures S7B, C**).

**Figure 3:**
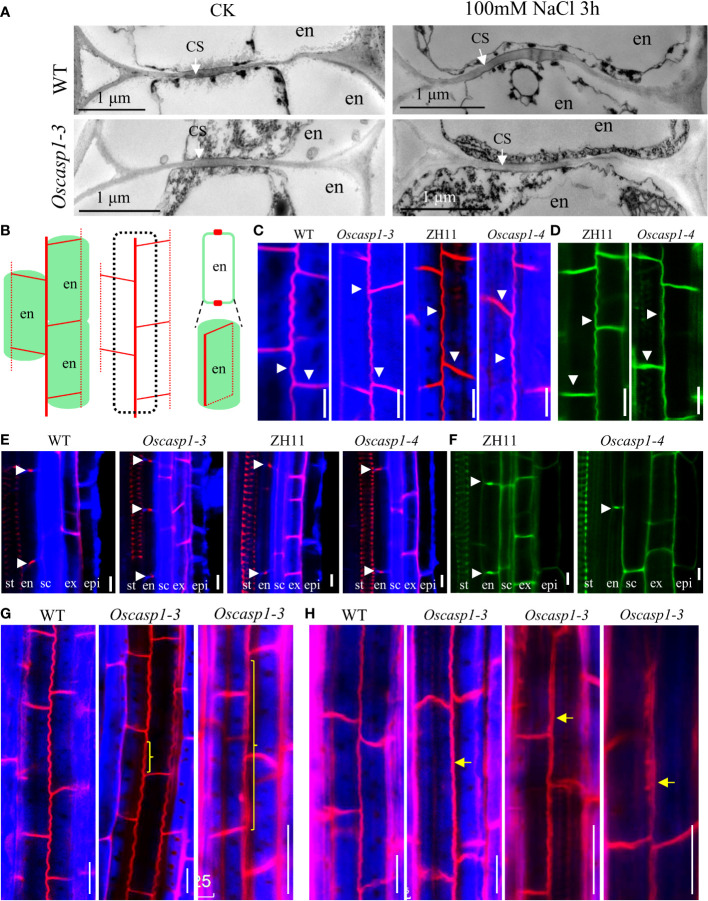
The Casparian strip structures of the roots. **(A)** The representative transmission electron micrographs of CS in primary roots of WT and *Oscasp1-3*, WT and *Oscasp1-3* treated with salt stress. These transverse sections were at 10 mm from primary root tip. **(B)** Cartoons of the observed lines and dots in an optical section, which represent a longitudinal and horizontal band in three dimensions, respectively. **(C, E**, **G, H)** SLRs stained with Basic Fuchsin and Calcofluor White in ClearSee solution. **(D, F)** SLRs stained with Auramine O in ClearSee solution. **(C, D)** The representative longitudinal and horizontal normal CS bands. **(E, F)** The representative CS dots. epi: epidermis; ex: exodermis; sc: sclerenchyma; en: endodermis. **(G, H)** The abnormal longitudinal CS bands in *Oscasp1-3*. Yellow curly brackets and arrows indicate abnormal CSs. **(G)** SLRs of 5-d-old seedling. **(C–F, H)** SLRs of 10-d-old seedling. Scale bar = 10μm.

To reveal the fine structures of CSs, we used a transmission electron microscope (TEM) to observe the sections of primary roots and found no difference between the *Oscasp1-3* mutant and WT (**Figure 3A**). We also checked the CSs after treatment with salt stress and still found no CS difference between the *Oscasp1-3* mutant and WT (**Figure 3A**). These results differ from the previous report (Wang Z. et al., 2019). Therefore, we observed CSs of the *Oscasp1-1* mutant using TEM too. Regrettably we did not uncover structural differences in CS between the *Oscasp1-1* mutant and Nipponbare (**Figure S8A**).

We assessed the expression levels of the genes involved in the formation of CS in the *Oscasp1-3* mutant and WT primary roots using RT-qPCR (**Table S1**). The result showed that the expression levels of *OsPER64* and *OsLOTR1* were significantly lower and the expression levels of *OsCASP3* and *OsCASP5* were significantly higher in WT than in *Oscasp1-3* (**Figure S8B**). These results suggest that the mutation of *OsCASP1* might enhance or accelerate lignin deposition due to high expression of *OsPER64* and *OsLOTR1* in primary roots (**Figure S8B**).

### Structural characteristics of small lateral roots in the wild type and *Oscasp1* mutant

In *Arabidopsis*, researchers determined CS structure and quantitatively described CS formation in very small roots by surface view of its autofluorescence (Naseer et al., 2012). Thus, we observed the structure of small lateral root (SLR) using these methods and found that there was no autofluorescence in SLR tips and no or very weak autofluorescence in SLR elongation zone of 6-d-old plants (**Figures S9A, B**), and stronger autofluorescence in most SLRs of 9-d-old and 15-d-old plants (**Figures S9C–I**). We could not determine where CS was in SLRs based on autofluorescence intensity, which was not stronger in CS than in other cells (**Figure S9J**). Moreover, almost all cells of primary roots displayed different intensities of autofluorescence, among which the sclerenchyma exhibited strongest fluorescence (**Figure S9J**). Older plants contain more lignin in the roots.

Basic Fuchsin was used for lignin staining and Auramine O was used for lignin and suberin staining (Ursache et al., 2018). Basic Fuchsin staining and Auramine O staining can clearly visualize the lignin deposition and CSs in endodermal cells in SLRs (**Figures 3B–F**). In the region close to SLR tips, we observed normal CS bands (**Figures 3C, D**) and normal CS dots (**Figures 3E, F**) in both the mutant and WT. In the region far from SLR tips, we discovered that approximately one-fifth of SLRs of 5-d-old seedlings and approximately one-half of SLRs of 10-d-old seedling contain abnormal CS in the *Oscasp1-3* mutant. These abnormal CSs either displayed wider bands with uneven lignin deposition (**Figures 3G** **, H**) or stronger bands with enhanced deposition (**Figure 3H**). The loss of OsCASP1 function may have reduced the control of lignin deposition.

**Figure 4:**
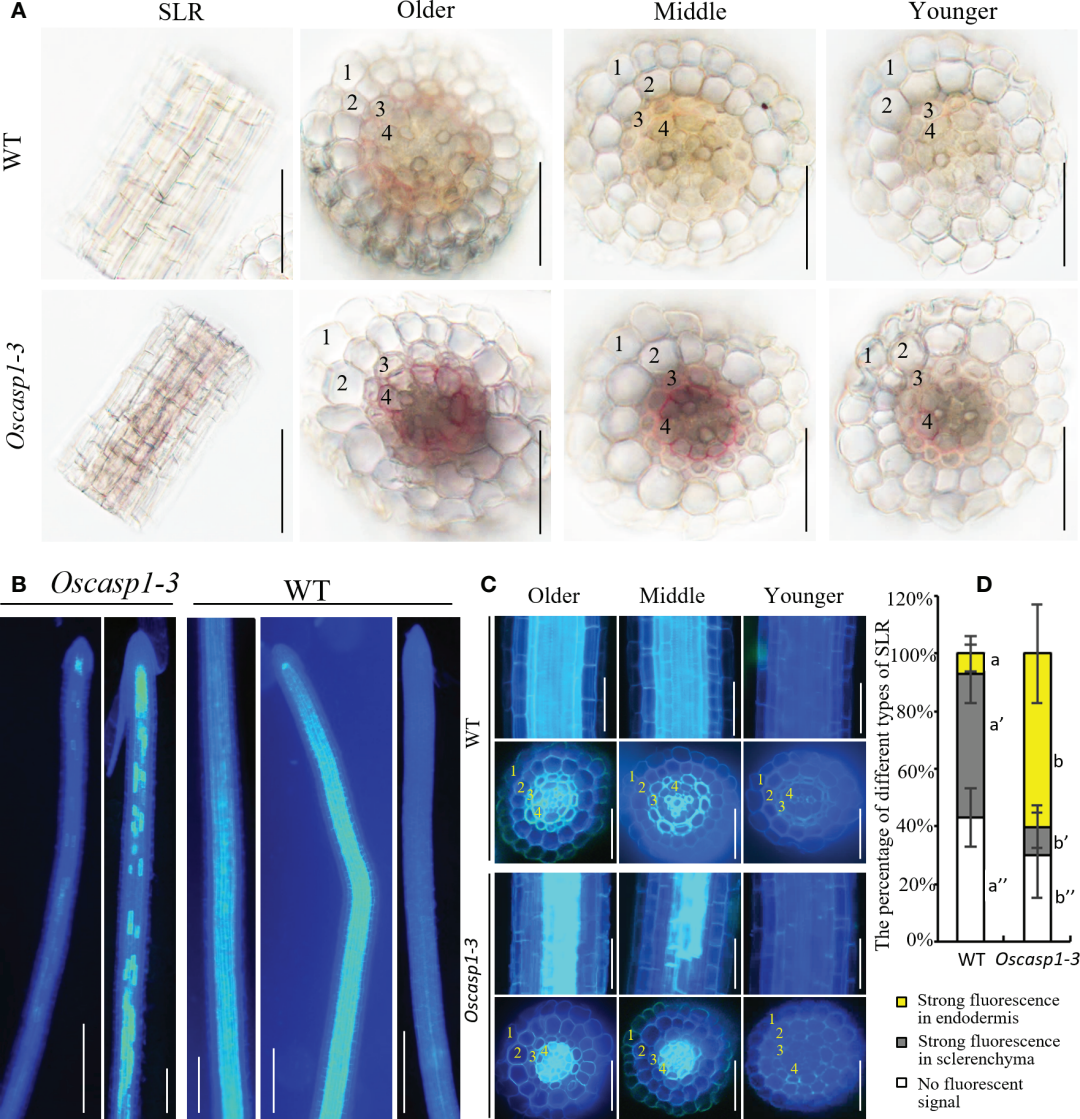
Characterization of the *Oscasp1-3* mutant and WT SLRs stained with berberine-aniline blue and phloroglucinol. **(A)** The representative SLR regions and cross-sections stained with phloroglucinol. Scale bar = 50 μm. **(B)** The representative images of whole SLRs stained with berberine-aniline blue. Scale bar = 100μm. **(C)** The representative SLR regions and cross-sections stained with berberine-aniline blue. Scale bar = 25 μm. **(D)** The percentage of different type of SLRs. Autofluorescence of cell walls is detected as blue. 1, epidermis; 2, exodermis; 3, sclerenchyma; 4, endodermis. Different letters denote significant differences in one-way ANOVA test (Tukey test p < 0.05) among different lines.

We also examined lignin deposition in SLRs stained with phloroglucinol, which specifically reacts with cinnamaldehyde end-groups of lignin to give a pink or fuchsia color. This method is less sensitive and more specific and quantitative than Basic Fuchsin staining (Pradhan Mitra and Loque, 2014; Hubbe et al., 2019). The result showed that the lignin deposition in the endodermis of the *Oscasp1-3* mutant was stronger than that of WT (**Figure 4A**). However, the lignin in the endodermis in the *Oscasp1-3* mutant SLRs was deposited not only in the radial walls but also in the outer peripheral walls of endodermal cells. Summarizing above results, the loss of OsCASP1 function leads to abnormal CS and enhanced lignin deposition in the endodermis.

### Loss of OsCASP1 function delays CS formation in SLRs

**Figure 5:**
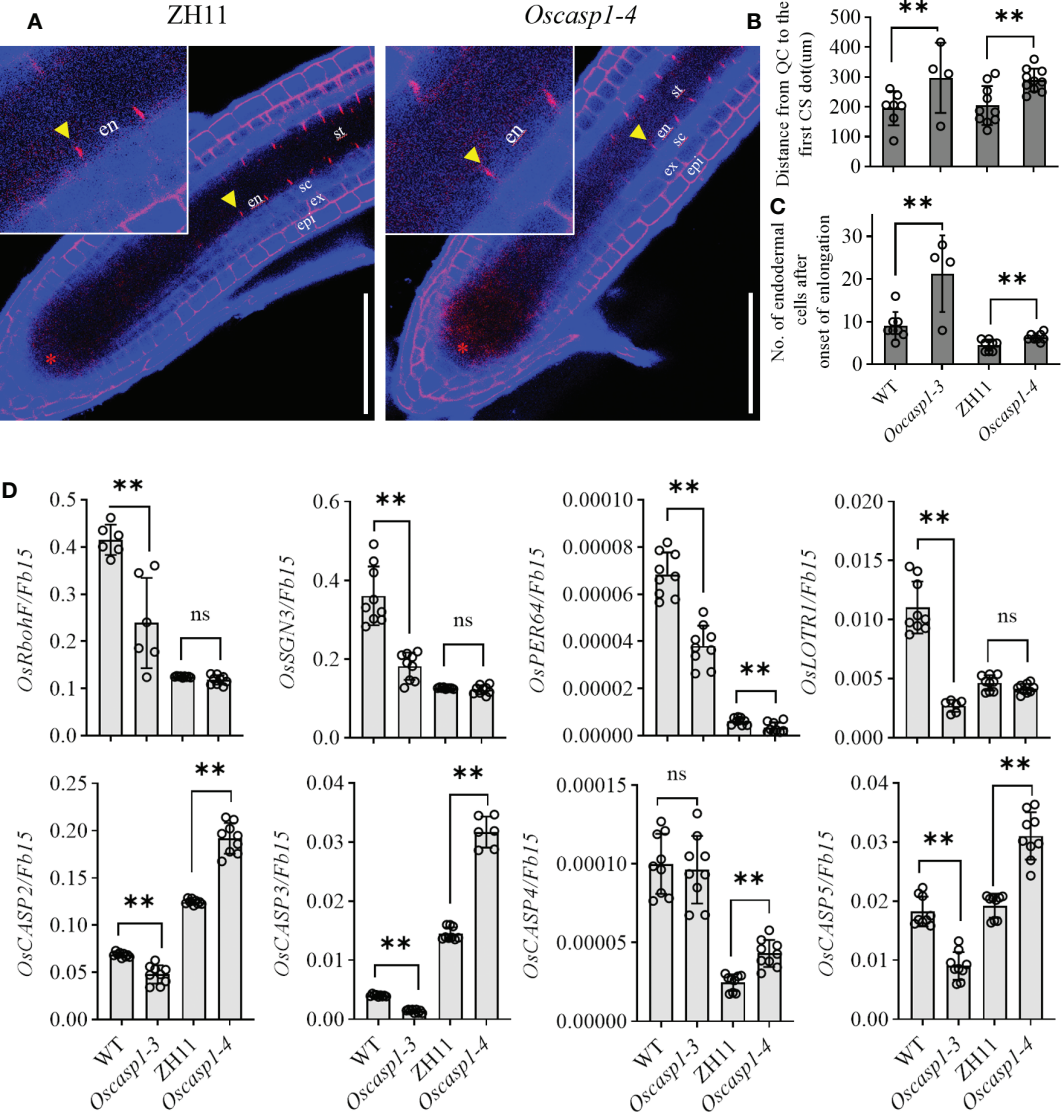
Significant delay of dot-like appearance of CS in *Oscasp1* mutants comparing with wild type plants. **(A)** The representative CS dots of the *Oscasp1-4* mutant and ZH11 SLRs stained with Basic Fuchsin and Calcofluor White in ClearSee solution. Yellow arrow heads indicate the first appearance of CS in endodermal cells. The red asterisks indicate the quiescent center (QC). st: stele; en: endodermis; sc: sclerenchyma; ex: exodermis; epi: epidermis. Scale bars = 75 μm. **(B)** The distance between QC and the first appearance of CS in endodermis were measured. **(C)** No. of endodermal cells between the first appearance of CS and “onset of elongation” were counted. “Onset of elongation” was defined as the zone where an endodermal cell was clearly more than twice its width. **(D)** Expression levels of the genes encoding the proteins that could participate in CS formation. Black circles represent independent replicates of specific values. Student’s T-test was used for statistical analysis, and ** represents statistically significant difference (p value < 0.01); ns: no significance. *Fb15* (Fiber protein 15) (*Os02g0175800*) was used as reference gene in the RT-qPCR analysis.

Mutations of most *Arabidopsis* genes regulating or participating in CS formation delay the CS formation (Doblas et al., 2017a). We wondered whether loss of OsCASP1 function would lead to delayed CS formation in SLRs. Thus, we selected some SLRs with similar diameter in the middle region of the primary root and observed the CS formation of the Basic Fuchsin-stained SLRs. The result showed that the appearance of the first CS in the *Oscasp1-3* and *Oscasp1-4* mutants was significantly delayed compared with their relevant WT (**Figures 5A–C**), and the difference between the *Oscasp1-3* and WT appeared to be greater than the difference between the *Oscasp1-4* and ZH11 (**Figure 5C**).

In order to explore the difference between the *Oscasp1-3* mutant and the WT, the *Oscasp1-4* mutant and ZH11, we assessed the expression levels of the genes involved in CS formation in SLRs using RT-qPCR (**Table S1**). With the exception of OsCASP4, the expression levels of these genes were significantly lower in the *Oscasp1-3* mutant than in its corresponding WT, while the expression levels of *OsCASP2*, *3*, *4*, and *5* were significantly higher in the *Oscasp1-4* mutant than in ZH11, and the expression level of *PER64* was significantly lower in the *Oscasp1-4* mutant than in ZH11 (**Figure 5D**). The genetic background may influence the expression of the genes involved in CS formation. Therefore, it is difficult to conclude that the delayed CS formation and abnormal CS in the *Oscasp1* mutant is due to altered expression of these genes. OsCASP1 may indirectly regulate CS formation.

### Characterization of SLRs revealed by berberine-aniline blue staining

An improved method for clearing with lactic acid saturated with chloral hydrate and staining with berberine-aniline blue was applied to visualize CS (Lux et al., 2005). We used this method to determine SLR structure and did not find CSs in SLR by surface observation (**Figure 4B**). We observed the cross-sections of the SLRs and discovered that the fluorescence of the sclerenchyma was much stronger than that of the endodermis in the WT. In contrast, the sclerenchyma in SLRs displayed much lower white-blue fluorescence in the *Oscasp1-3* mutant (**Figure 4C**). We observed that many SLRs of the *Oscasp1-3* mutant contained patchy white-blue zones from endodermal cells, whereas only a few SLRs of WT did. Some endodermal cells in patchy regions of the *Oscasp1-3* mutant displayed strong white-blue fluorescence, while the SLRs of the WT displayed continuous white-blue fluorescence, which was strong or weak (**Figure 4B**). Younger SLRs, which were closer to the primary root tip, exhibited lower white-blue fluorescence, and older SLRs, which were closer to seeds, showed continuous white-blue fluorescence in WT and more patchy white-blue fluorescence in the *Oscasp1-3* mutant (**Figure 4C**). Fluorescence intensity depended on the positions of the SLRs in the primary root. However, since berberine-aniline blue can stain both lignin and suberin, it is difficult to distinguish whether the white-blue fluorescence arises from lignin or suberin (Brundrett et al., 1988). We have not observed patchy deposition of lignin in SLRs of the *Oscasp1-3* mutant and WT (**Figure S10**), therefore, we speculated that the patchy fluorescence in *Oscasp1-3* and strong fluorescence of the sclerenchyma in WT could come from the suberin deposition.

### The small lateral roots of the *Oscasp1* mutant display ectopic suberin deposition

The previous report indicated that the *Oscasp1-1* mutant showed priority accumulation of suberin in primary roots by checking root cross-sections at different root positions (Wang Z. et al., 2019). We repeated the experiment in the same way, but it is very difficult to obtain a consistent result with the method due to considerable variation among roots. This may be because suberin deposition is very sensitive to ionic change and other environment factors (Barberon et al., 2016).

**Figure 6:**
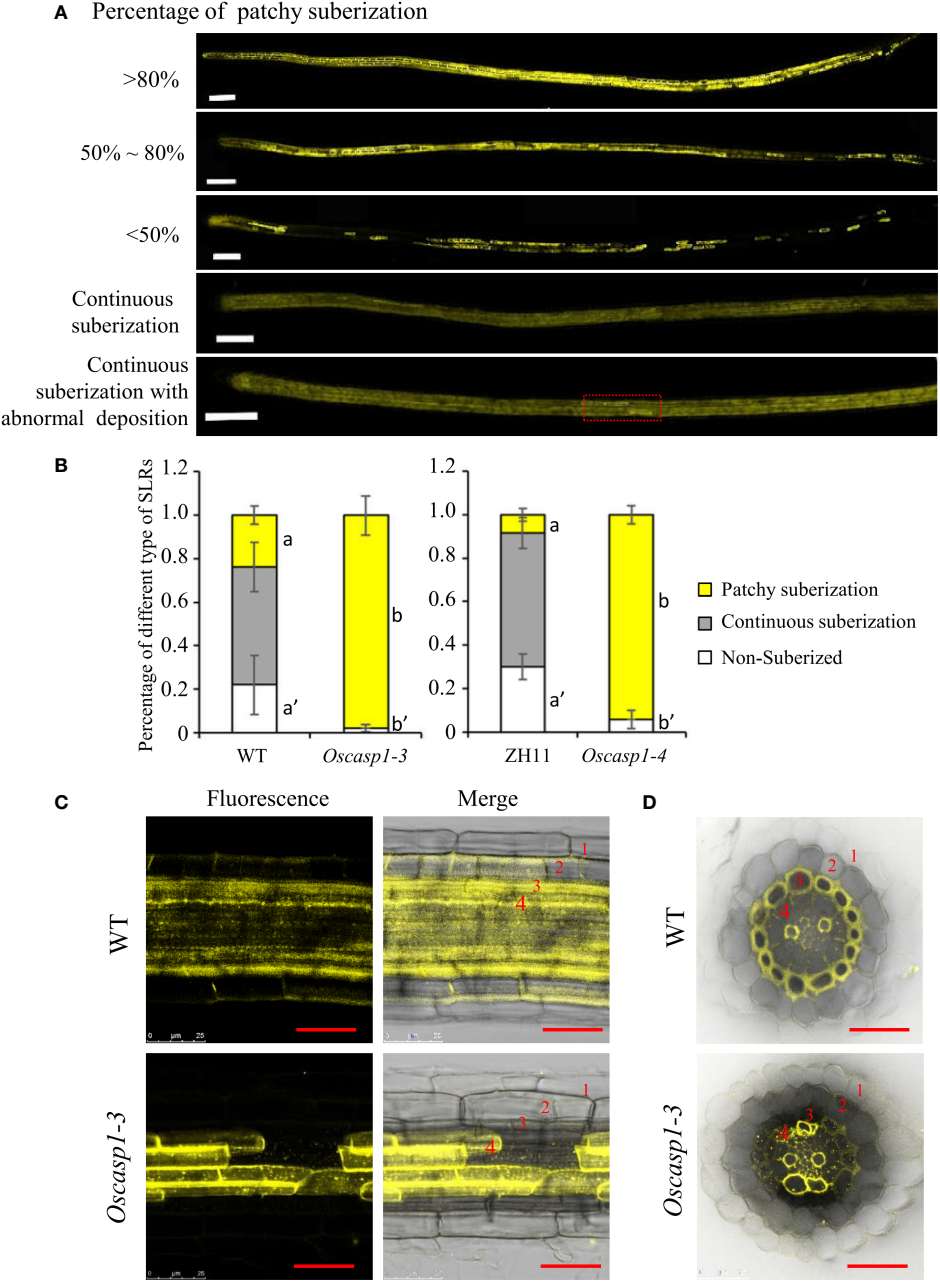
The patterns of suberin deposition of the small lateral roots. **(A)** The patterns of suberin deposition in small lateral roots of the WT and *Oscasp1-3* mutant, which were obtained with laser confocal microscopy. Scale bar = 100μm. **(B)** The percentage of small lateral roots of each deposition pattern. Different letters denote significant differences in one-way ANOVA test (Tukey test p < 0.05) among different lines. **(C)** The representative pattern of suberin deposition in the WT and *Oscasp1-3*. Scale bar = 25μm. **(D)** Representative transverse sections from small lateral roots. Scale bar = 25μm. 1, epidermis; 2, exodermis; 3, sclerenchyma; 4, endodermis.

The above result hinted that the patchy fluorescence in the mutant SLRs stained with berberine-aniline blue arose from suberin accumulation. We then evaluated suberin deposition in SLRs with Fluorol Yellow 088. The results showed that the patterns of suberin deposition depended on the position of SLRs in the primary root. The newborn lateral roots near the primary root tip (2~3 cm behind the root apex) usually had no suberin deposition, and the SLRs without suberin deposition occurred more frequently in the WT than in the mutant (**Figure 6B**). The SLRs farther from the primary root tip in WT usually exhibited uniform staining and some SLRs contained a few strongly staining cells (**Figure 6A**). This result indicated that suberin deposition was evenly distributed along the SLRs in the WT. However, most SLRs in *Oscasp1-3* showed abnormal suberin deposition and an uneven distribution along the SLRs, and suberization increased along with root growth (**Figure 6A**). To further determine in which cell layer the suberin is deposited, we observed the cross-sections of the SLRs after FY088 staining. The result showed that strong fluorescence signal was mainly concentrated in the sclerenchyma cell wall and weak fluorescence signal was in the radial wall of endodermal cells in WT (**Figures 6C, D**). Nevertheless, we detected strong fluorescence signal in the endodermal cell wall and weak in the sclerenchyma cells of the *Oscasp1-3* mutant (**Figures 6C, D**). This is in good agreement with the result of berberine-aniline blue staining. These results suggest that the loss of OsCASP1 function changes the pattern and location of suberin deposition in rice SLRs.

**Figure 7:**
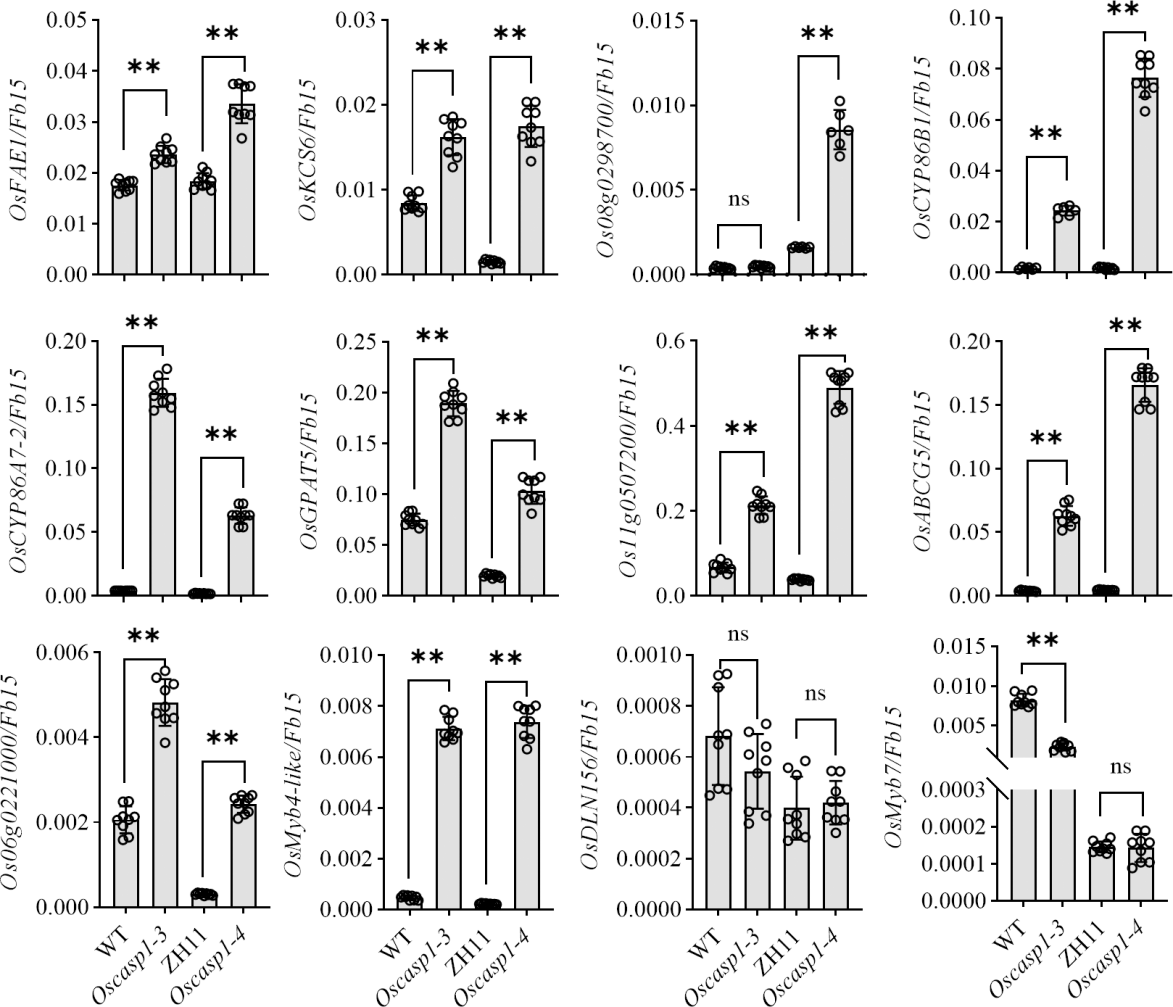
Expression levels of the genes involved in suberin synthesis. Student’s T-test was used for statistical analysis, and ** represents statistically significant difference (p value < 0.01); ns: no significance.

**Figure 8:**
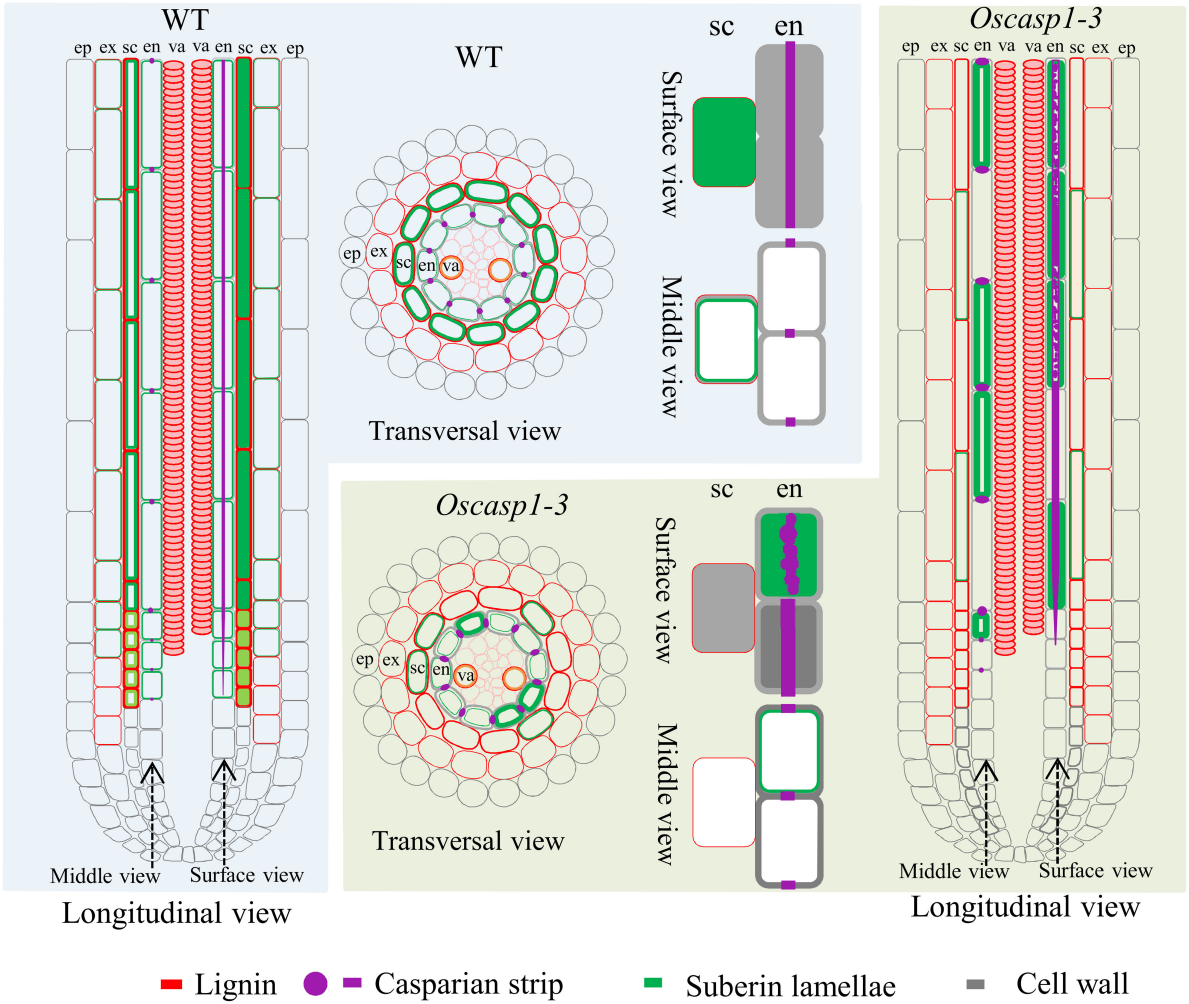
Schematic longitudinal and transversal views of SLRs and single cells showing characteristics of SLRs and the differences between WT and *Oscasp1-3* in rice. Lignin and suberin depositions in the endodermis and sclerenchyma are represented by red and green lines, respectively. CSs are represented by purple dots and lines, and cell walls are represented by grey lines. ep, epidermis; ex, exodermis; sc, sclerenchyma; en, endodermis; va, vascular tissue.

In *Arabidopsis*, many genes regulating or participating in suberin synthesis were revealed (**Table S1**). Since many of the characterized suberin biosynthetic enzymes and their encoding genes exhibit conserved functionality across species, we assessed the expression levels of these genes that are orthologs of *Arabidopsis* genes involved in suberin synthesis (Woolfson et al., 2022)(**Table S1**). The expression levels of these genes were significantly higher in the two *Oscasp1* mutants than in their corresponding WTs, and the expression level of *Os08g0298700* was significantly higher in *Oscasp1-3* than the WT (**Figure 7**). We also determined the expression levels of four Myb transcription factors that could regulate the expression of suberin biosynthetic genes (**Table S1**) and found that the expression levels of two Myb TF genes (*Os06g0221000* and *OsMyb4-like*) were significantly higher in the two mutants and that expression level of *OsMyb7* was significantly lower in *Oscasp1-3* (**Figure 7**). These results further confirmed that the loss of OsCASP1 function enhanced the expression of most related genes and resulted in ectopic suberin deposition. Finally, we summarized the above results and presented a schematic diagram to reveal the characteristics of SLRs and the differences between WT and *Oscasp1-3* in rice (**Figure 8**).

### The apoplastic barrier function to PI

Propidium iodide (PI) was widely used as an apoplastic tracer to reveal the functional apoplastic barrier in roots of *Arabidopsis* (Alassimone et al., 2012) and rice (Wang Z. et al., 2019; Wang Z.et al., 2022), thus we applied this substance in our study. However, we occasionally found that high concentration of PI easily penetrated CSs into the steles of rice roots and that CSs could not completely block the PI penetration. Therefore, we investigated whether PI could be used as an apoplastic tracer in rice root research. We first examined the permeability of primary roots to PI and uncovered that lower PI concentration took more time and higher concentration required less time to enter into the stele of rice roots. Younger root might show higher permeability than older root (**Figure S11**). WT roots could not block, but could hinder PI penetration into stele (**Figure S11**). Since PI was firstly used in *Arabidopsis*, we wondered whether higher concentration or longer staining time was able to overcome obstruction of CS into root stele. We stained the roots of 5-d-old seedling and discovered that *Arabidopsis* roots could completely block the entry of PI into stele regardless of concentration and staining time (**Figure S12**). These results suggest that the permeability and CS structure of rice roots are different from those of *Arabidopsis*.

We also examined the permeability of SLRs and found that different locations of primary roots and different zones of SLR showed different permeability to PI. SLRs farther from the primary root tip usually exhibited more retardation to PI, and the nascent SLR completely lost PI block (**Figure S13A**). We examined SLRs of eight 6-d-old and 7-d-old seedlings. The number of SLRs that could not prevent PI from entering varied greatly from plant to plant (**Figure S13**). Furthermore, we found that environmental condition appeared to affect the permeability of SLRs to PI, which resulted in the difficulties in quantitative analysis.

**Figure 9:**
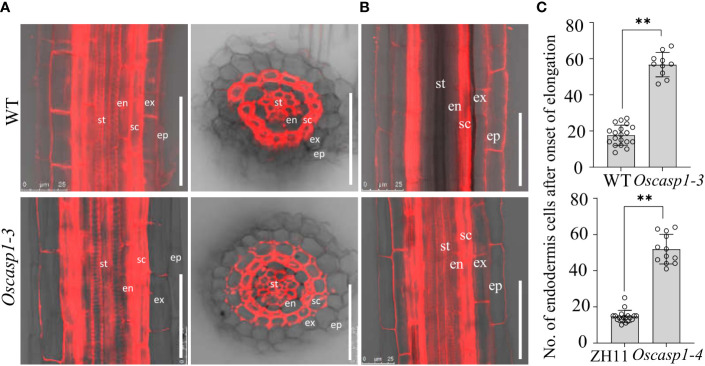
PI penetration in the *Oscasp1-3* mutant and WT roots. **(A)** Representative images of *Oscasp1-3* and WT SLRs showed the areas that lose barrier to PI in both of the mutant and WT. SLRs were incubated with 10 μg/ml PI for 10 minutes. **(B)** The area of WT SLR blocked PI penetration into stele. st, stele; en, endodermis; sc, sclerenchyma; ex, exodermis; ep, epidermis. Scale bar = 50 μm. **(C)** Quantification of PI penetration into the stele quantified as number of endodermal cells from the first fully expanded cell in wild type. Student’s T-test was used for statistical analysis, and ** represents statistically significant difference (p value < 0.01); ns: no significance.

We examined PI permeability of mutant roots. The primary roots of WT appeared to exhibit lower permeability than that of the *Oscasp1-3* mutant (**Figure S11**), and SLRs of the *Oscasp1-3* mutant were more sensitive to staining time (**Figures S13C, D**). We selected some SLRs that were in the middle of the primary root and had a similar diameter and compared the cellular distance from the first cell at which diffusion barrier appears to the first fully expanded cell in SLRs. The result showed that the cellular distances of the *Oscasp1-3* and *Oscasp1-4* mutants were significantly longer than that of WTs (**Figure 9**). Compared with **Figure 5**, we observed a radical difference between the appearance of the first CS and the block of PI diffusion. The block of PI diffusion appeared much later (**Figure 9**) and could not be attributed to CS structure. Summarizing above results, CSs in rice roots could not block PI into steles.

### The *Oscasp1-3* mutant displays defective ion homeostasis and different sensitivities to different nutrient stresses

**Figure 10:**
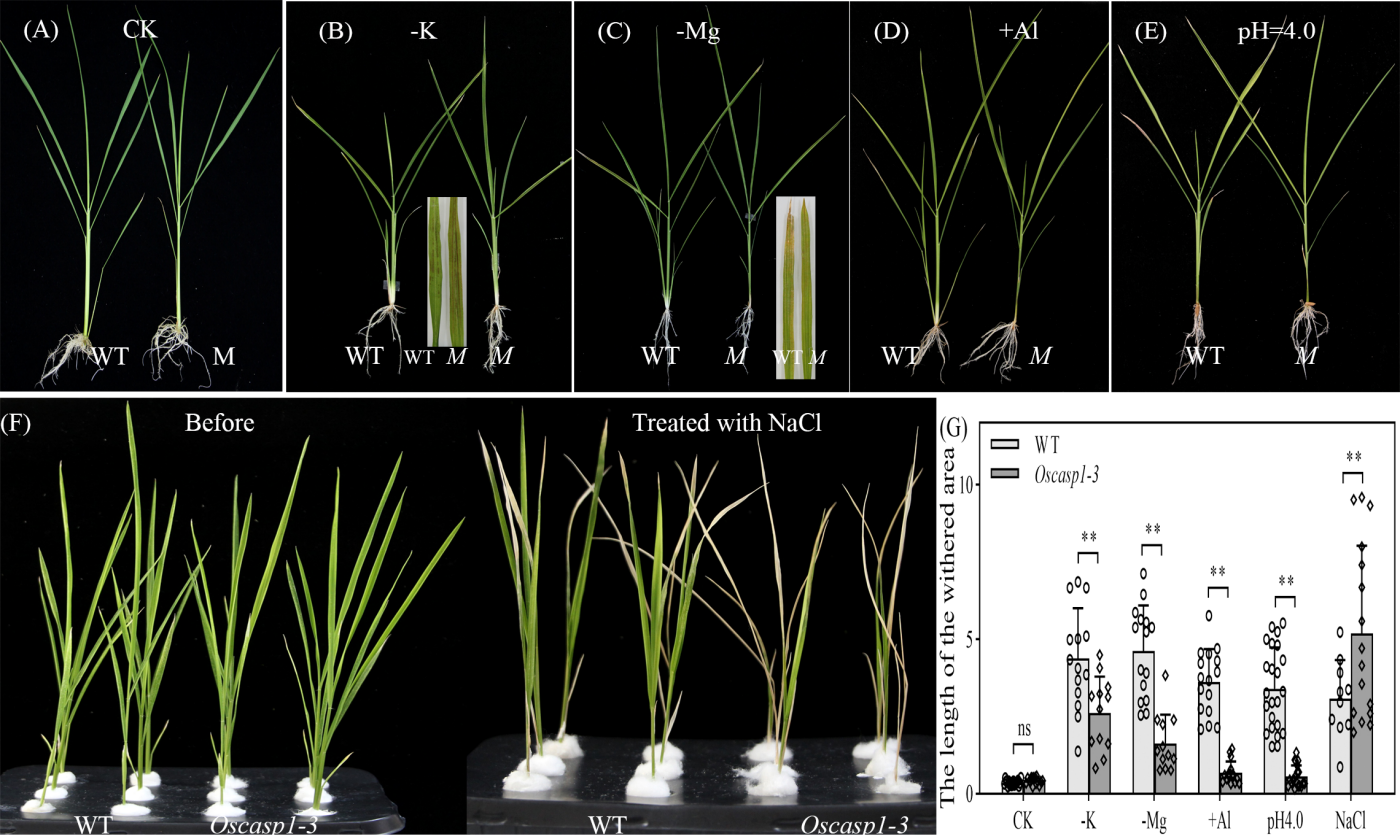
Phenotypes of WT and *Oscasp1-3* mutant plants grown in various media with elemental mineral imbalances. **(A)** In complete medium. **(B)** In medium without potassium. **(C)** In medium without magnesium. **(D)** In medium with 100 µM AlCl_3_. **(E)** In medium with a low pH value (pH = 4.0). **(F)** In medium with 100 mM NaCl. **(G)** The length of the withered leaf area of these seedlings grown in various media. Student’s T-test was used for statistical analysis, and ** represents statistically significant difference (p value < 0.01); ns: no significance. M: *Oscasp1-3*.

Loss of OsCASP1 function changed suberin deposition in SLRs and probably altered the ion permeability of the mutant. We measured the content of 9 metal elements in leaves at the tillering stage, where a small number of dead cells visible to the naked eye appeared. The result showed that the *Oscasp1-3* mutant had significantly higher concentrations of iron, manganese, and sodium and lower concentrations of potassium and arsenic (**Figure S14**). The *OsCASP1* mutation altered ion uptake in the root, and the previous report has also indicated that the shoots of the *Oscasp1-1* mutant display defective nutrient homeostasis (Wang Z. et al., 2019). We speculated that the disorder of ion homeostasis in plants resulted in the mutant phenotype, and then we examined the growth of *Oscasp1-3* plants in nutrient-poor solution in a climate incubator and discovered that the mutant displayed distinct phenotypes under different stress conditions. There was no visible leaf cell death in the *Oscasp1-3* mutant in complete medium. The mutant was insensitive to the deficiency of phosphorous, iron, or nitrogen and to a high concentration of phosphorous or iron (data not shown), and there were slight differences in the leaves between *Oscasp1-3* and WT in medium without potassium, magnesium, or with aluminum (**Figures 10A–D** **, G**, and **Figures S15A–C**). Compared to the *Oscasp1-3* mutant, the WT exhibited more tillers and fewer and shorter roots in potassium-free medium; the WT showed earlier senescence of lower leaves and more tillers in medium without magnesium or with aluminum (**Figures 10B–D** **, G** and **S15B, C**); and the WT was earlier senescence to the medium with a low pH value (pH = 4.0) (**Figures 10E, G**, and **Figure S15D**). In addition, the mutant showed more curled and dry leaves in the medium with cadmium (**Figures S15E**) and was more sensitive to high concentrations of NaCl relative to the WT (**Figures 10F–G**). These results suggested that the *Oscasp1-3* mutant exhibited different sensitivity to different nutrient stresses, which could be relevant to the composition of mineral ions in the mutant plant.

## Discussion

### Characterization of lignin deposition of rice roots and the role of OsCASP1 in CS formation

Our study showed that the expression of *OsCASP1* gene is concentrated at the SLR tip and is strongly expressed after NaCl treatment especially in the steles (**Figure 2**). Its mutation delays CS formation (**Figure 5**) and leads to uneven lignin deposition in the endodermis (**Figure 3**) and ectopic suberin deposition in SLRs (**Figure 6**). Comparing with previous reports, our results differ in the following points (Wang Z. et al., 2019; Wang Z. et al., 2022). The first concerns the CS structure of the *Oscasp1* mutant in primary roots. We employed multiple approaches to observe CS structures of the primary roots and did not observe significant difference between the mutants and their corresponding WTs (**Figures 3A**, **S6**, **S7**, and **S8A**). Previous reports indicated that more CSs at 10 mm from apex and almost all CSs at 15 mm, and 20 mm from apex exhibit significantly broader and thicker bands in the *Oscasp1* mutant and that the “broad CS” is abnormal or defective (Wang Z. et al., 2019; Wang Z. et al., 2022). Unfortunately, we were not able to reproduce their results nor find more “broad CS” in the mutant (**Figure S7**). We also noticed that the pattern of lignin deposition revealed with Basic Fuchsin staining is different from that with berberine-aniline blue and phloroglucinol (**Figures S6**, **S7**). There is an area in the radial walls of endodermal cells that cannot be stained by Basic Fuchsin. In fact, it is difficult to determine where the CS is according to staining results. Wang Z. et al. (2019) thought that one of lignin bands is CS, but the evidence was insufficient (Wang Z. et al., 2019). In order to determine whether there is a CS difference between the *Oscasp1* mutant and WT, we used TEM to clearly observe structure of CS, which remains attached to the cell membrane after plasmolysis. We did not find difference between the mutants and their corresponding WTs, including between the *Oscasp1-1* mutant and Nipponbare used in the previous study (Wang Z. et al., 2019) (**Figures 3A** and **S7**). Summarizing the above results and previous reports, we speculate that the loss of OsCASP1 function enhances or accelerates lignin deposition (**Figure S7A**) (Wang Z. et al., 2019; Wang Z. et al., 2022). Furthermore, in addition to the radial wall, we observed lignin deposition in outer peripheral and stele-facing walls of endodermal cells and in the cell wall of cortical cells (**Figure S7**). The deposition pattern of lignin in rice roots is different from that in *Arabidopsis* roots.

The second concerns the localization of OsCASP1, which was found to specially accumulate in the CS-forming region of endodermal cells in rice roots by immunostaining with GFP antibody (Wang Z. et al., 2019; Wang Z. et al., 2022). This conclusion is different from our results (**Figures 2** and **S5**). Carefully reading these reports, we found that the relevant experiments lacked negative controls. We repeated the experiment using Nipponbare (without *GFP* gene), observed cross-sections at 7 mm, 10 mm, and 20 mm from primary root tips, and found green fluorescence and red fluorescence at CS-forming region of endodermal cells (**Figure S16**). The green fluorescence could come from the autofluorescence of CSs, and the red fluorescence came from non-specific binding (**Figure S16**). The green fluorescent pattern is similar to that in the previous reports (Wang Z. et al., 2019; Wang Z. et al., 2022). Thus, it is necessary to add a negative control for immunostaining experiment in the previous study to eliminate false positives. Moreover, *OsMyb36a*, *OsMyb36b*, and *OsMyb36c* are strongly expressed in root tips like *OsCASP1* (**Figures 2A** and **S5A**), whose expression is not only concentrated in the endodermis. OsMyb36a, OsMyb36b, and OsMyb36c can bind to the promoter of *OsCASP1* and directly regulate its expression (Wang Z. et al., 2022). It is strange that Wang Z. et al. (2019, 2022) cannot detect the OsCASP1 accumulation in other tissues, such as steles and sclerenchyma. However, our results showed that the expression pattern of *OsCASP1* is more consistent with that of *OsMyb36a, OsMyb36b*, and *OsMyb36c* compared to the previous report (Wang Z. et al., 2022) Overexpression of *OsMyb36a* accelerates CS formation, whereas co-mutation of *OsMyb36a/b/c* delays CS formation in endodermis (Wang Z. et al., 2022). If the conclusion that OsCASP1 directly regulate CS formation is correct, the consistency of expression pattern between *OsCASP1* and *OsMyb36s* could better explain the conclusion that OsMYB36 modulates CS formation through regulating *OsCASP1* expression.

The third concerns PI penetration, which was used to detect the permeability of CS in *Arabidopsis* (Naseer et al., 2012; Hosmani et al., 2013; Barberon, 2017). However, our finding suggests that rice roots can hinder, but not prevent, the entry of PI into the stele (**Figures 9A** and **S11**, **S13**). Thus PI was not suitable for detecting CS integrity in rice root. The PI permeability of rice roots should be different from that of *Arabidopsis*.

Since thicker primary roots of rice seedlings are not suitable for whole-mount observation, we pay more attention to CS structure of SLRs. After treatment with ClearSee solution and staining with Basic Fuchsin and Calcofluor White, whole-mount observation of SLR can obtain clear CS structure. We discovered some abnormal CS bands and delayed CS formation in the mutant SLRs (**Figures 3G, H**, **5B, C**). Compared with *Arabidopsis* roots, the first appearance of CS in rice is earlier than that in *Arabidopsis* (**Figure 5C**) (Naseer et al., 2012). Most abnormal CSs displayed uneven lignin deposition (**Figures 3G, H**), and strangely, abnormal CSs were mostly found in the region far from SLR tips. The lignin deposition in endodermis may be out of control in the *Oscasp1* mutant. Combining the above results and previous reports (Wang Z. et al., 2019; Wang Z. et al., 2022), we propose that OsCASP1 can regulate CS formation, but whether OsCASP1 can form a scaffold with itself or other OsCASPs in CSD for CS biosynthesis needs more evidences.

### The suberin deposition in the *Oscasp1* mutant SLRs

The recent report indicated that the loss of OsCASP1 function enhances suberin deposition in primary roots of the *Oscasp1* mutant by observing cross-sections. Unfortunately, the conclusion lacks statistical support (Wang Z. et al., 2019). We observed many cross-sections from different zones of many primary and large lateral roots and could not obtain consistent results to support the difference. Since suberin deposition of the endodermal cells is influenced by environmental factors and the location of the cells in roots (Barberon et al., 2016; Barberon, 2017), statistical analyses are necessary to determine the deposition pattern of suberin. However, it is difficult to quantify the onset of suberin accumulation and the difference between the *Oscasp1* mutant and WT based on cross-sectional results. Our study suggests that *Oscasp1* mutations result in heterogeneous enhancement of suberin deposition in the endodermis and loss of suberin deposition in the sclerenchyma, not only strong suberization through observing SLRs (**Figures 4**, **6**). The deposition patterns of suberin in rice roots are different from that in *Arabidopsis* roots. Moreover, the plasticity of suberin is a major means of coping with nutrient stress by regulating their uptake or their retention in the vasculature (Barberon et al., 2016). Suberin deposition is induced by drought stress, salt treatment, and waterlogging condition and is important for growth under waterlogged conditions in rice (Krishnamurthy et al., 2009; Krishnamurthy et al., 2011; Ranathunge et al., 2011; Shiono et al., 2014). Thus, defective suberin lamellae in SLR could impair rice adaptation to growth environments.

### Observation of SLRs treated with ClearSee solution accelerates progress in anatomical and developmental investigations

In *Arabidopsis*, the number of endodermal cells from the first fully expanded cell can be counted to quantitatively describe the formation of CS, the deposition of suberin, and permeability to PI using whole-mount method (Hosmani et al., 2013). *Arabidopsis* rootlets have a simple structure, including the epidermis, cortex (with only one cell layer), endodermis, and stele, and the autofluorescence of CS in the endodermis is strong and clearly visible (Roppoloet et al., 2011; Naseer et al., 2012). Rice rootlets are more complex than *Arabidopsis* and are not suitable for whole-mount observation. SLRs in rice have a simple structure too, consisting of epidermis, exodermis, sclerenchyma, endodermis, and stele (Rebouillat et al., 2009), in which lignin is deposited and displays autofluorescence except the epidermis (**Figure S9**). ClearSee solution significantly diminishes autofluorescence. In combination with confocal microscope, this treatment allows researcher to observe thicker tissues and improves image quality after the sample was stained with fluorescent dye.

To date, most knowledge about root structure and development has been gained through microtomy. However, the laborious nature of thin sectioning, the problem of obtaining the desired section plane, and the difficulty of obtaining complete series of sections limit the application of the technique. However, thick samples are often difficult to visualize without sectioning due to autofluorescence and tissue complexity. Here, we chose SLR as the study object and used the ClearSee technique to study CS formation and suberin deposition in SLRs, which reduces the time and cost of embedding/sectioning and difficulty of the research, and discovered that *Oscasp1* mutations resulted in delayed CS formation, abnormal lignin, and suberin deposition in SLRs. Comparing with previous reports, these findings deepens our understanding of OsCASP1-mediated CS formation and suberin deposition in rice roots.

### OsCASP1 represents a novel regulative way of CASPs

The *OsCASP1* gene is highly expressed at the SLR tip, and its expression is highly induced by salt treatment especially in the steles, whereas *AtCASPs* are specially expressed in the endodermis (Roppolo et al., 2011). *AtCASPL* genes are expressed in tissues other than the endodermis, such as *AtCASPL2A1* in the lateral root tip, *AtCASPL5B1* in immature and differentiated trichomes in leaves, and *AtCASPL1F1* in anthers (Roppolo et al., 2014). *AtCASPL4C1* gene is widely expressed in a variety of organs and is cold-inducible, and the mutant shows elevated tolerance to cold stress (Yang et al., 2015). *AtCASPL1D2* is exclusively expressed in suberized endodermal cell and could regulate suberin deposition induced by NaCl stress (Champeyroux et al., 2019). These results suggest that AtCASPL proteins have various functions. OsCASP1 has a different function from AtCASPs and can appear similar to some AtCASPLs.

SLRs, in which lignin and suberin deposition are regulated by OsCASP1 (**Figures 4**, **6**), are more sensitive to the perception of environmental conditions than other root types. The result gives a hint that OsCASP1 plays an important role in abiotic stress responses. The deposition patterns of lignin and suberin in rice roots, which are different from that of *Arabidopsis* roots, could be the reason why rice is adapted to the aquatic environment. The *Oscasp1* mutant is more sensitive to upland condition (Wang Z. et al., 2019) and shows different tolerances to different nutrient stresses (**Figures 10** and **S15**). These results suggest that OsCASP1 could play an important role in the adaptation to the growth conditions.

## Data availability statement

The original contributions presented in the study are included in the article/**Supplementary Material**. Further inquiries can be directed to the corresponding author.

## Author contributions

XS conceived and designed the experiments, wrote the manuscript, and took part in some experiments. HX completed the map-based cloning of *Oscasp1-3* gene. HX and QW did complementation test and CRISPR/Cas9 of OsCASP1. XY performed electron microscopy, lignin and suberin deposition analysis, and RT-qPCR. XY and QW performed histological analysis and hydroponic experiment. KL and YG performed field experiments and management. All authors contributed to the article and approved the submitted version.
