# Supplementary material for: Rice OsCASP1 orchestrates Casparian strip formation and suberin deposition in small lateral roots to maintain nutrient homeostasis
Source: Front Plant Sci. 2022 Dec 19;13:1007300. doi: 10.3389/fpls.2022.1007300 (PMC9807177; doi:10.3389/fpls.2022.1007300)
Supplement: Supplementary file 1 [file DataSheet_1.pdf]

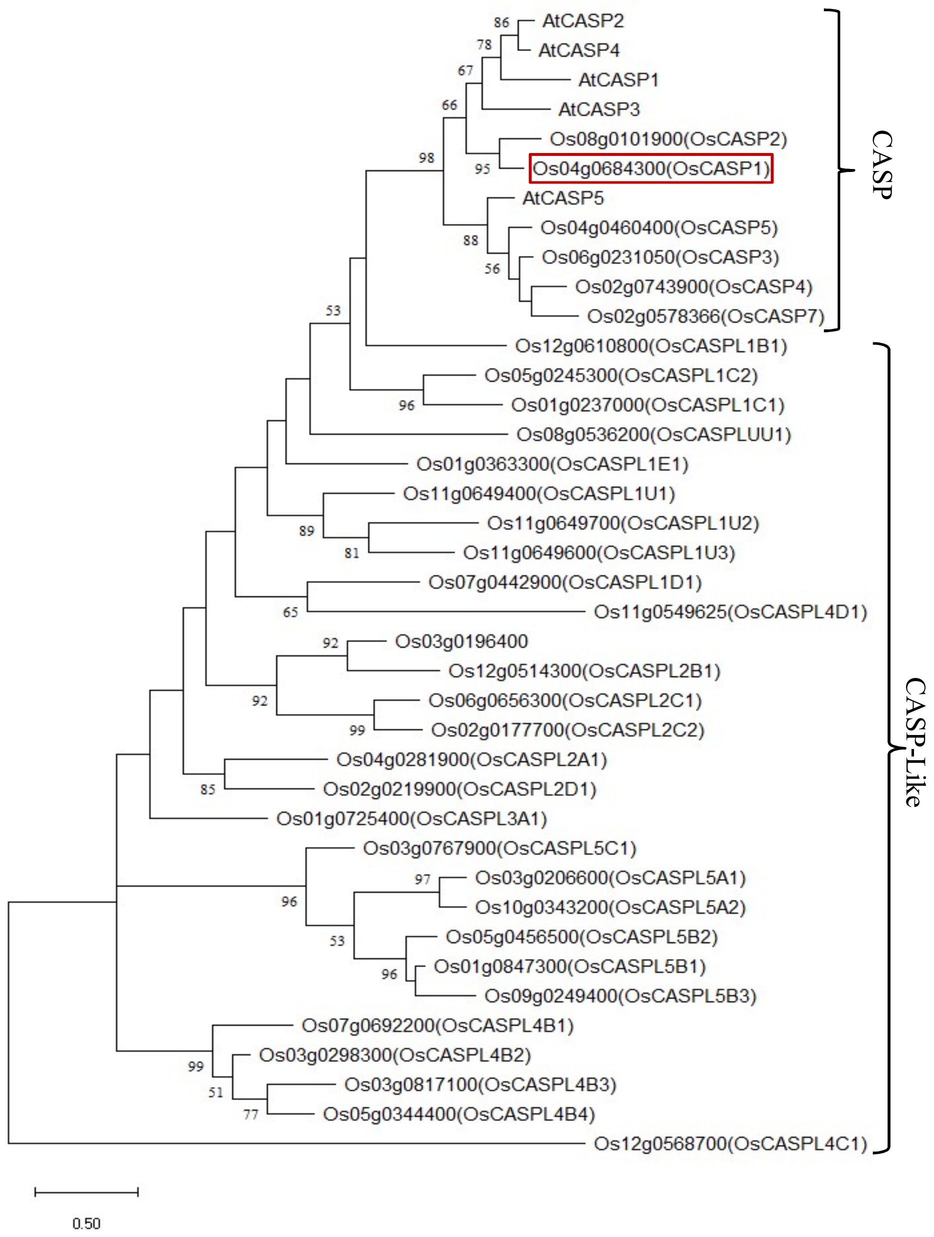

Figure S1. The maximum-likelihood tree of OsCASP1 homologs with their protein sequences. The scale bar indicates the simple matching distance. The alignment and phylogenetic tree were constructed with MEGAX (http://megasoftware.net/). The numbers for interior branches indicate the bootstrap values for 500 replications.

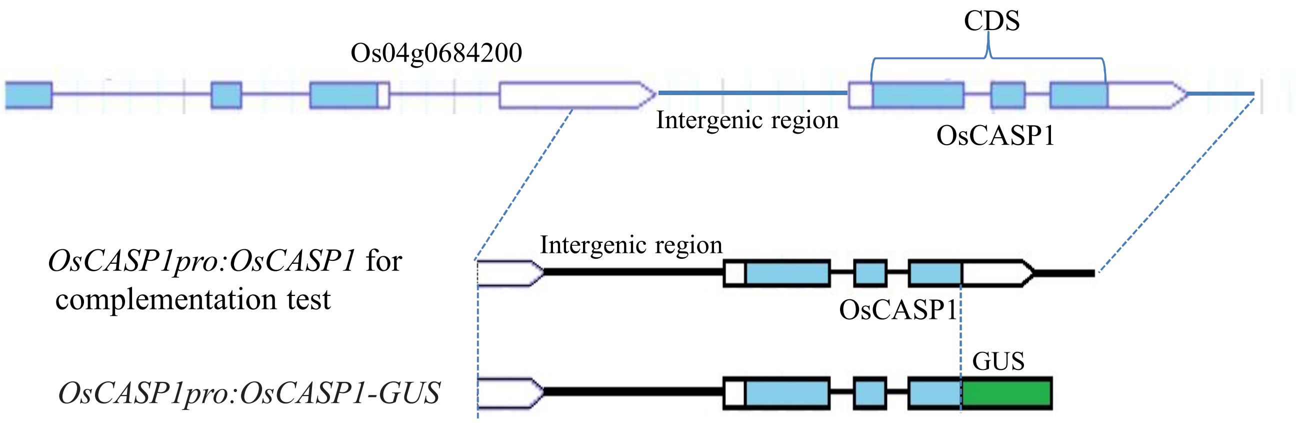

Figure S2. Models of the constructs for complementation test and tissue-specific expression of *OsCASP1*.

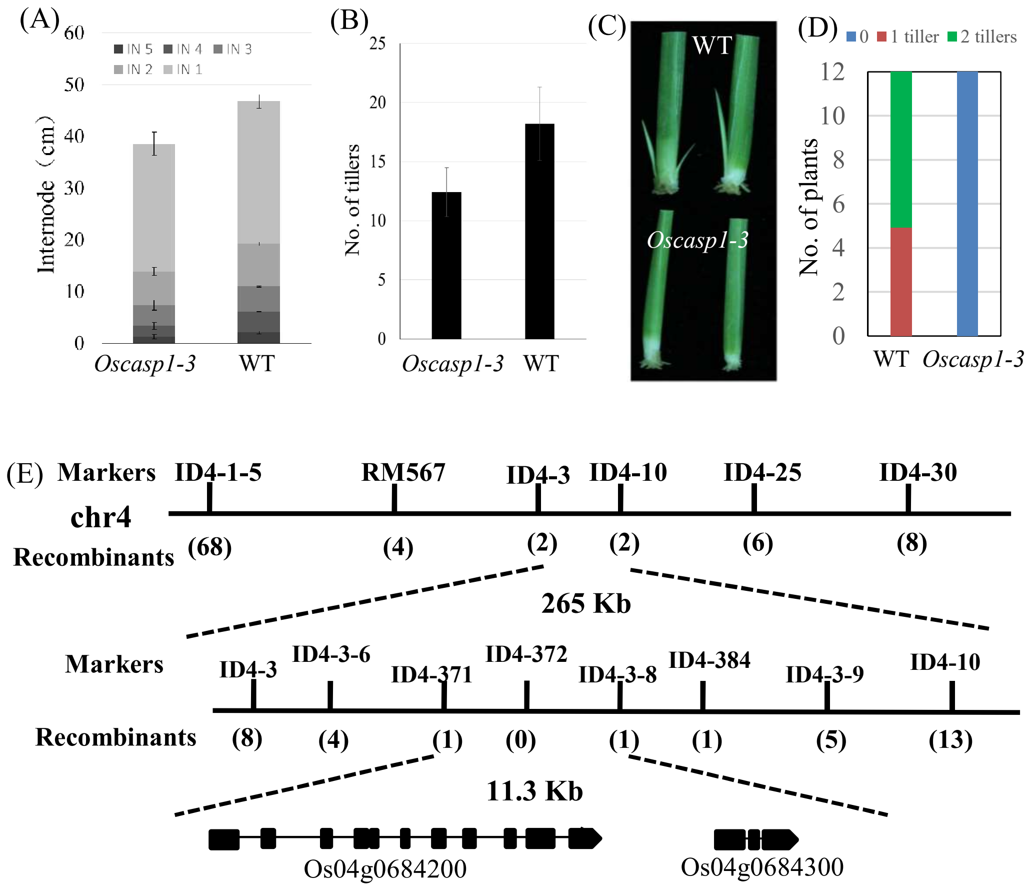

Figure S3. The phenotypes of the WT and *Oscasp1-3* mutant and characterization of *Oscasp1-3*.
(A) Internode length. IN = internode. (B) The tiller number of the WT and *Oscasp1-3* mutant at the heading stage. (C) and (D) The tiller phenotype of the seedling at 33-d-old. The emergence of tiller in the *Oscasp1-3* mutant was delayed. (E) The *Oscasp1-3* locus was fine-mapped on chromosome 4. The numbers of recombinants are shown in brackets.

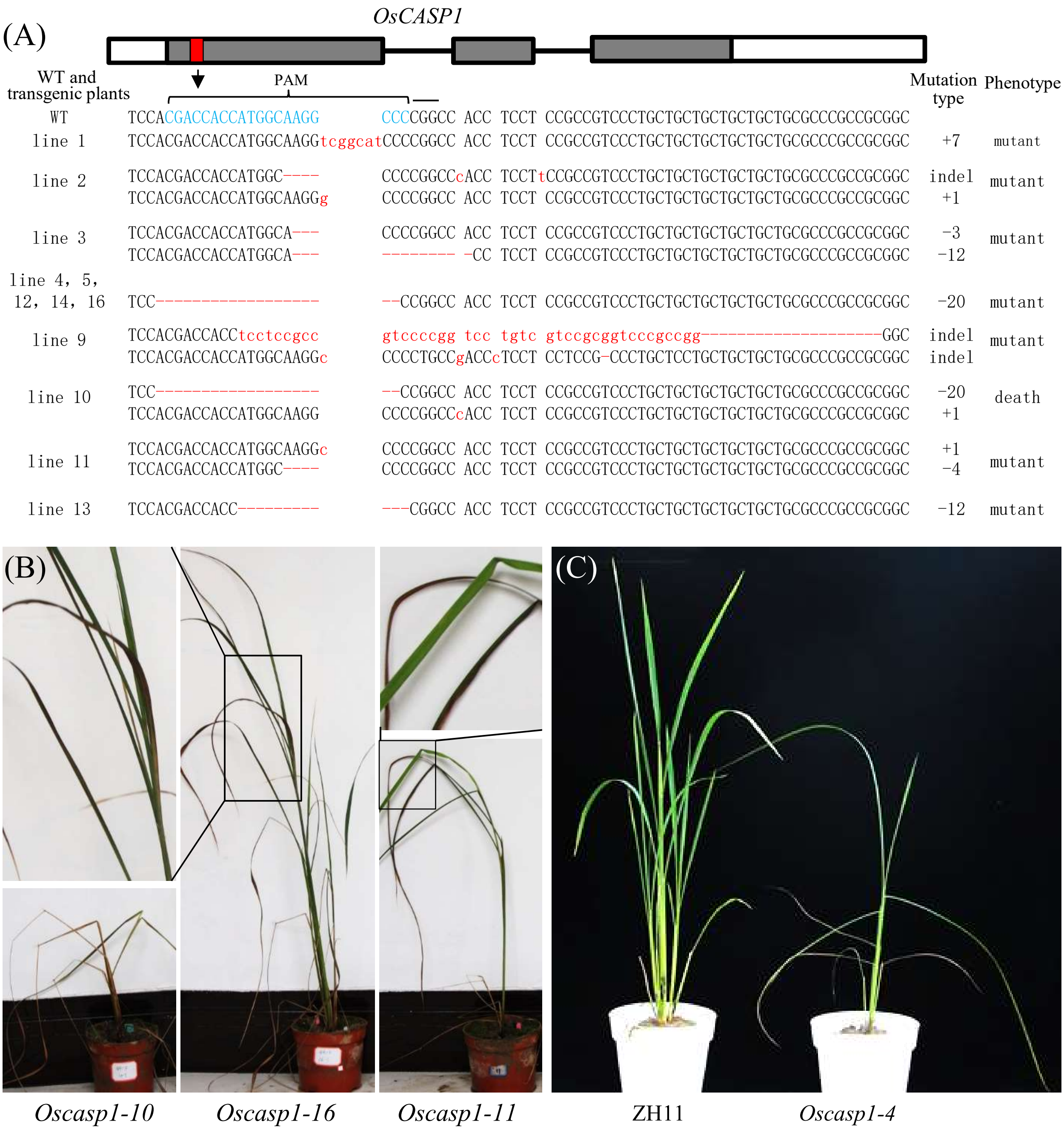

Figure S4. The genotype and phenotype of CRISPR/cas9 mutants. (A) Schematic map of *OsCASP1* gene and sequence alignment of the sgRNA target region showing altered bases in different mutant lines. The target region of the sgRNA was indicate with arrow, and the heterozygous transgenic plants with different mutant sites were marked with rectangles. (B) The typical phenotypes of CRISPR/cas9 transgenic plants. (C) The typical phenotype of the *Oscasp1-4* mutant, which is homozygous progeny of *Oscasp1-4* (T0 generation) and contains a 20bp deletion in *OsCASP1* gene.

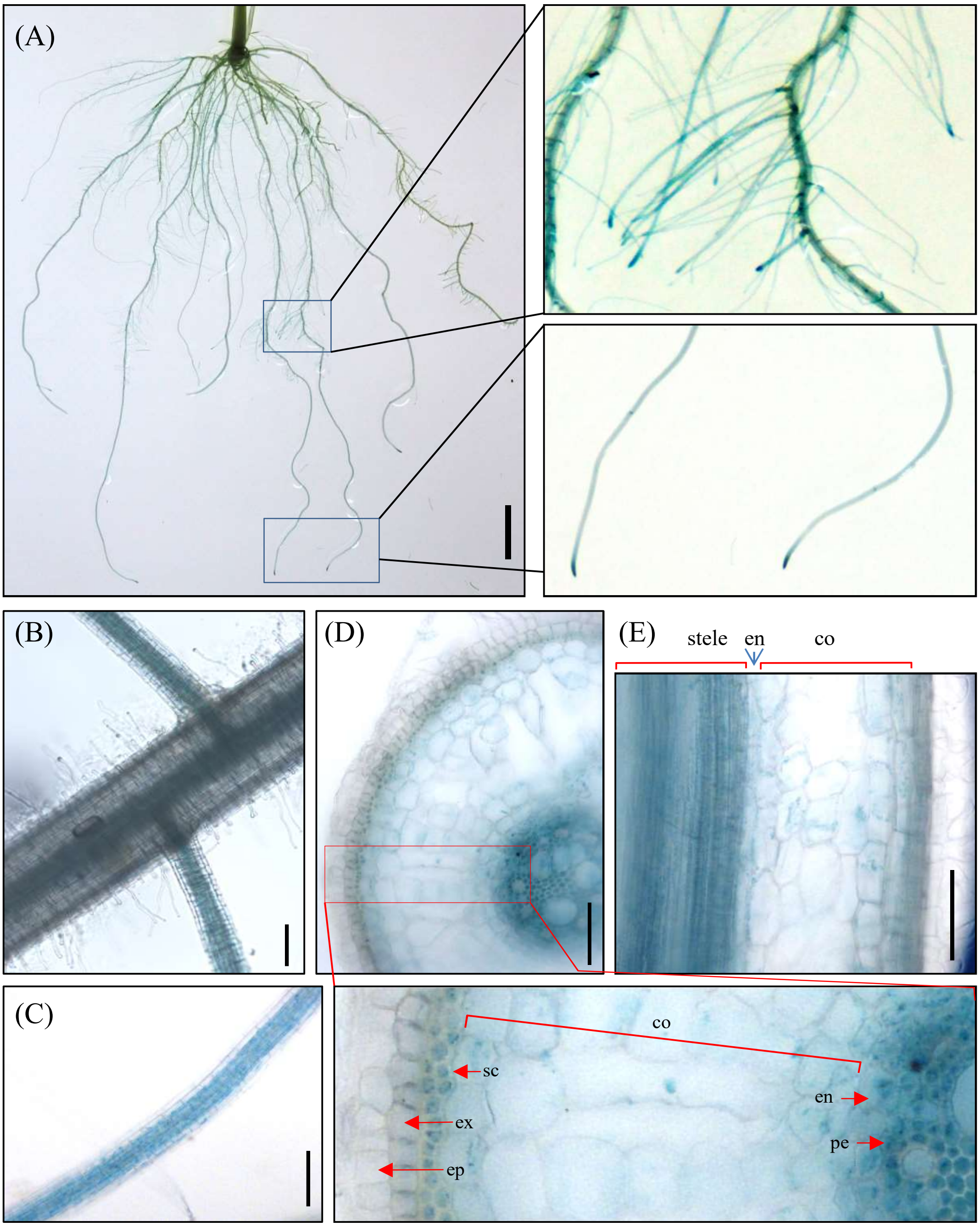

Figure S5. The localization of *OsCASP1pro:OsCASP1-GUS* expression after salt treatment. (A) Whole roots treated with NaCl. (B) Crown root with lateral roots. (C) Lateral root. (D) Transection of the crown root. (E) Longitudinal section of the crown root. ep, epidermis; ex, exodermis; sc, sclerenchyma; co, cortex; en, endodermis; pe, pericycle. Scale bar: (A) 1cm; (B), (C), (D) and (E) 0.1cm.

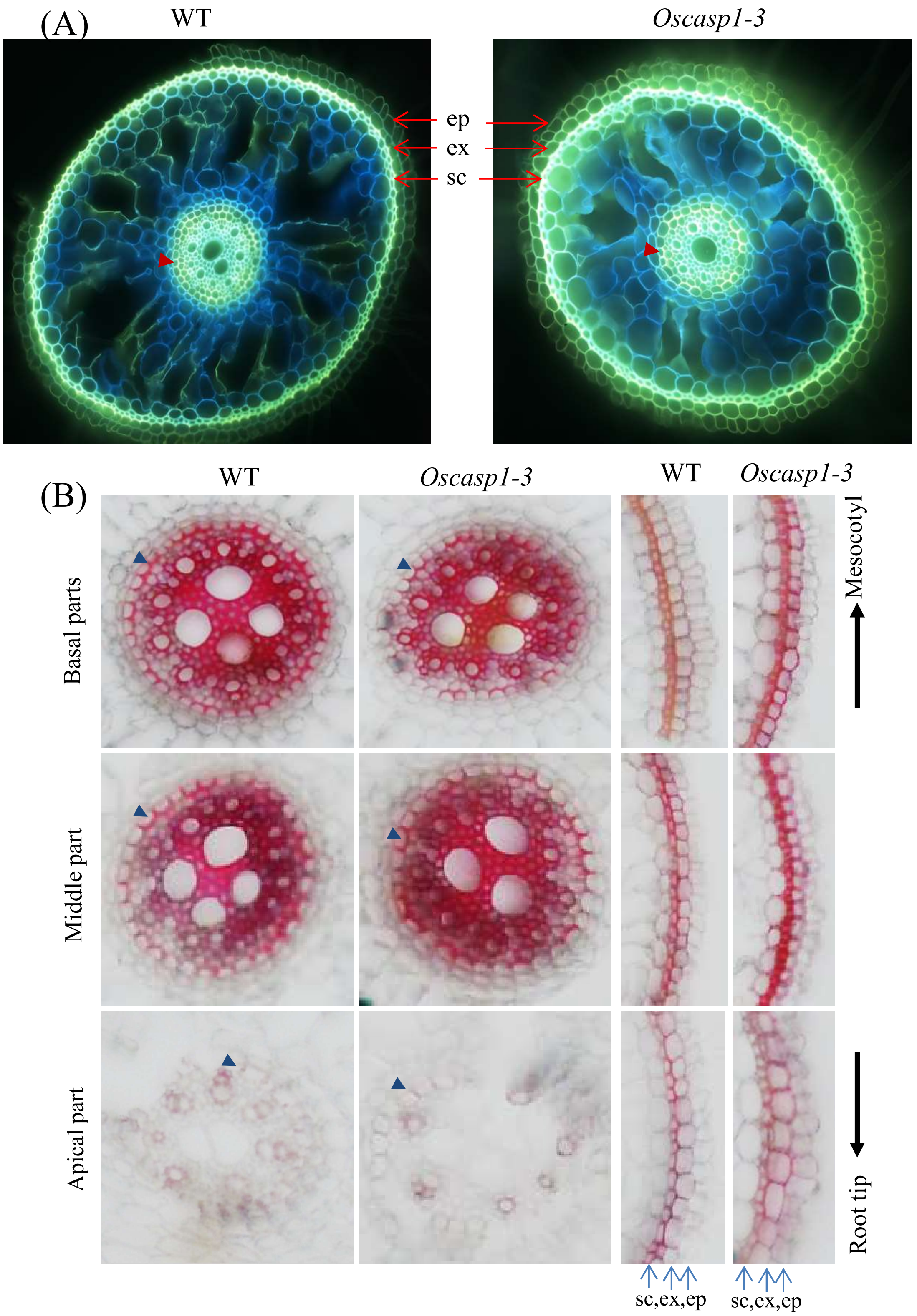

Figure S6. The representative cross-sections at different zones in primary roots stained with berberine-aniline blue and phloroglucinol. (A) Staining with berberine-aniline blue. Autofluorescence of cell walls is detected as blue. Arrow head indicates CS at endodermis. (B) Staining lignin with phloroglucinol. Arrow head indicates endodermis. ep, epidermis; ex, exodermis; sc, sclerenchyma.

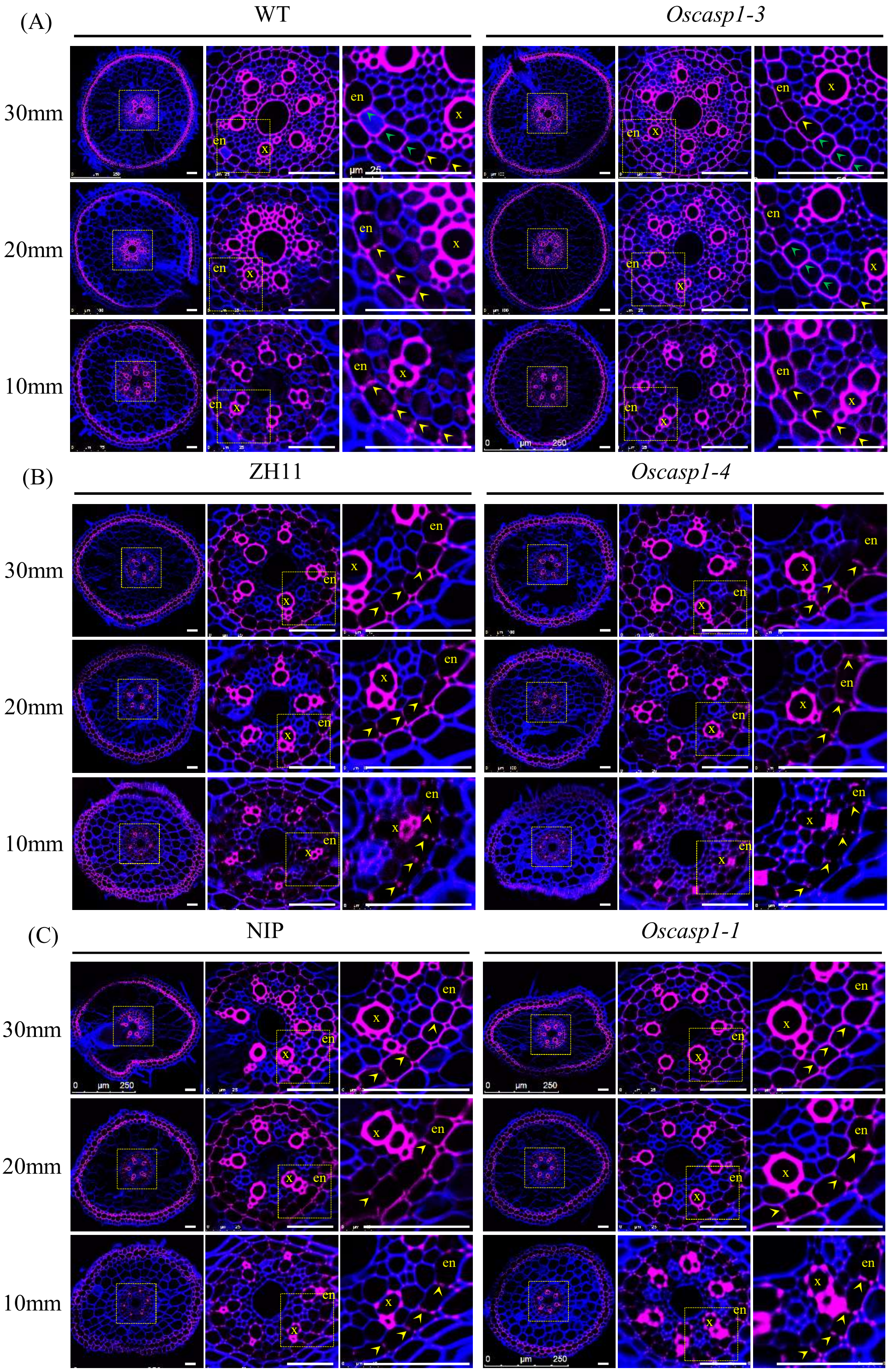

Figure S7. The representative cross-sections of the primary root stained with basic fuchsin. The cross-sections at 10 mm, 20 mm, and 30 mm from root tips were observed. (A) The *Oscasp1-3* and WT. (B) *Oscasp1-4* mutant and Zhonghua 11 (ZH11). (C) The *Oscasp1-1* mutant and Nipponbare (NIP). The yellow boxed areas are magnified in their right images. en: endodermis; x: is xylem. Arrows indicate possible CSs based on the previous report (Wang et al. 2019b). (A) (B) (C) Scale bar = 50 μm. ; x: is xylem.

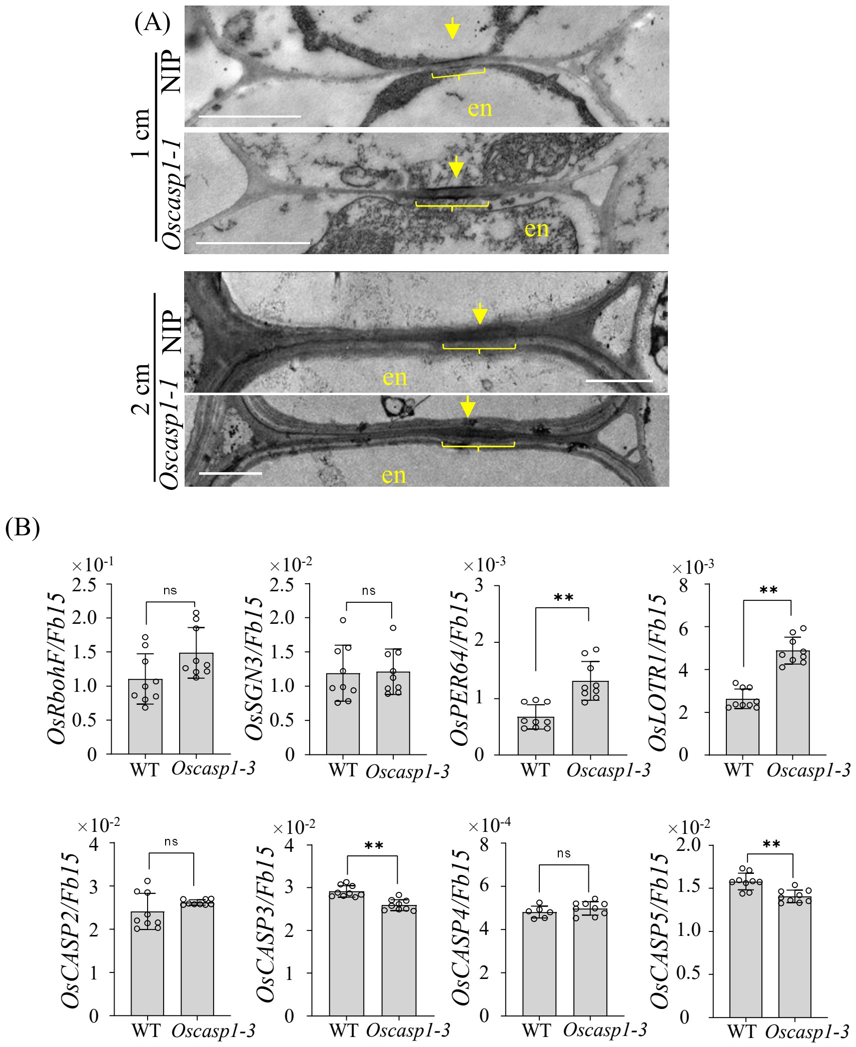

Figure S8. (A) The transverse sections 10 mm and 20 mm from root tips. NIP: Nipponbare; en: endodermis, and yellow curly brackets indicate the range of CS. (B) Expression levels of the genes associated with CS formation in primary roots of 10-d-old seedlings. The 1 cm long root tips of the primary roots were used for RT-qPCR. Student's T-test was used for statistical analysis, ns: no significance; **: P<0.01. *Fb15* (Os02g0175800) was used as reference gene in the RT-qPCR analysis.

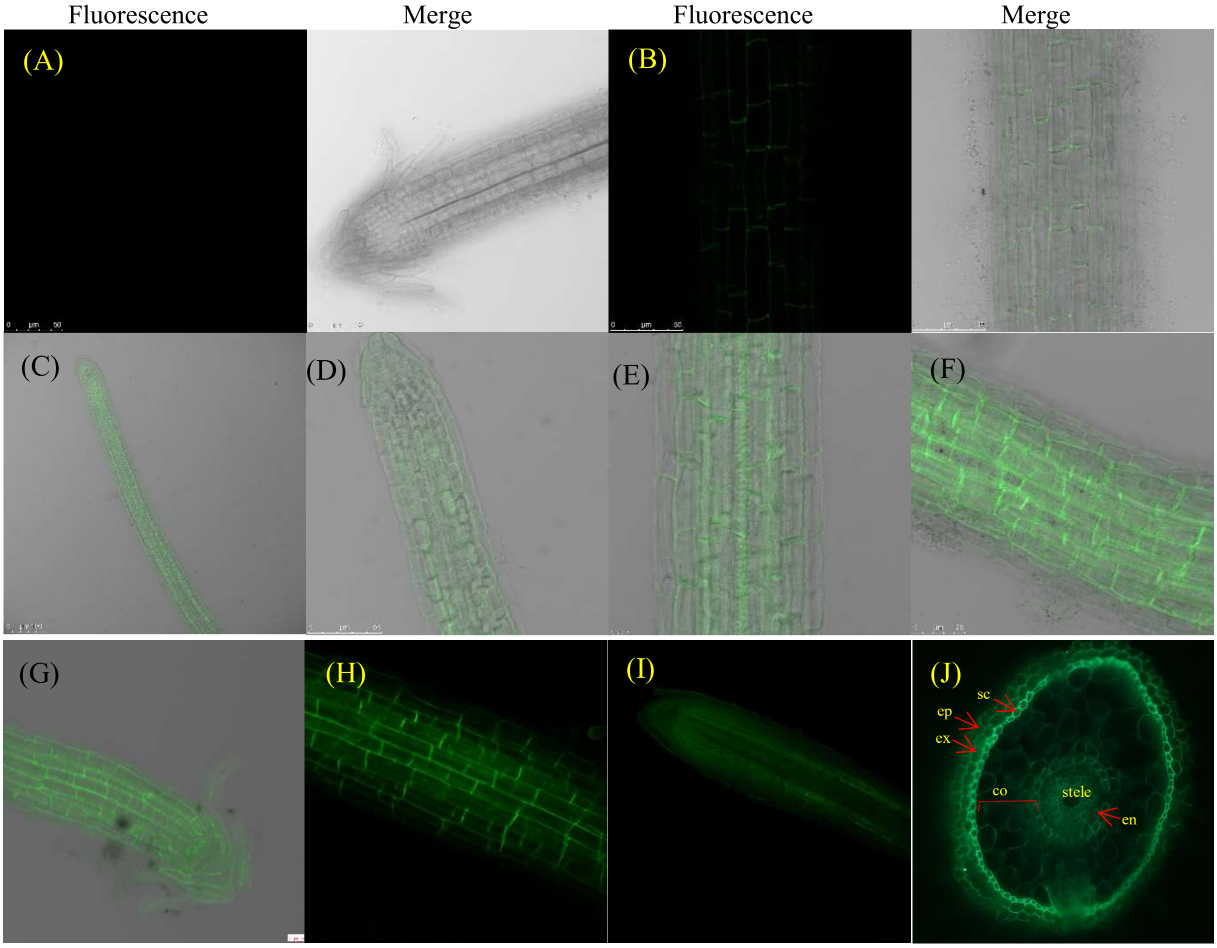

Figure S9. Autofluorescence in rice roots were detected at excitation of 488nm wavelength and 100% HyD gain (default value) with a Leica Confocal Microscope. (A) and (B) Small lateral roots of 6-d-old plant. (C), (D), (E) and (F) Small lateral roots of 9-d-old plants with different focuses, respectively. (G), (H) and (I) Small lateral roots of 15-d-old plants with different focuses, respectively. (J) The cross-section of primary root of 14-d-old plant. ep, epidermis; ex, exodermis; sc, sclerenchyma; co, cortex; en, endodermis.

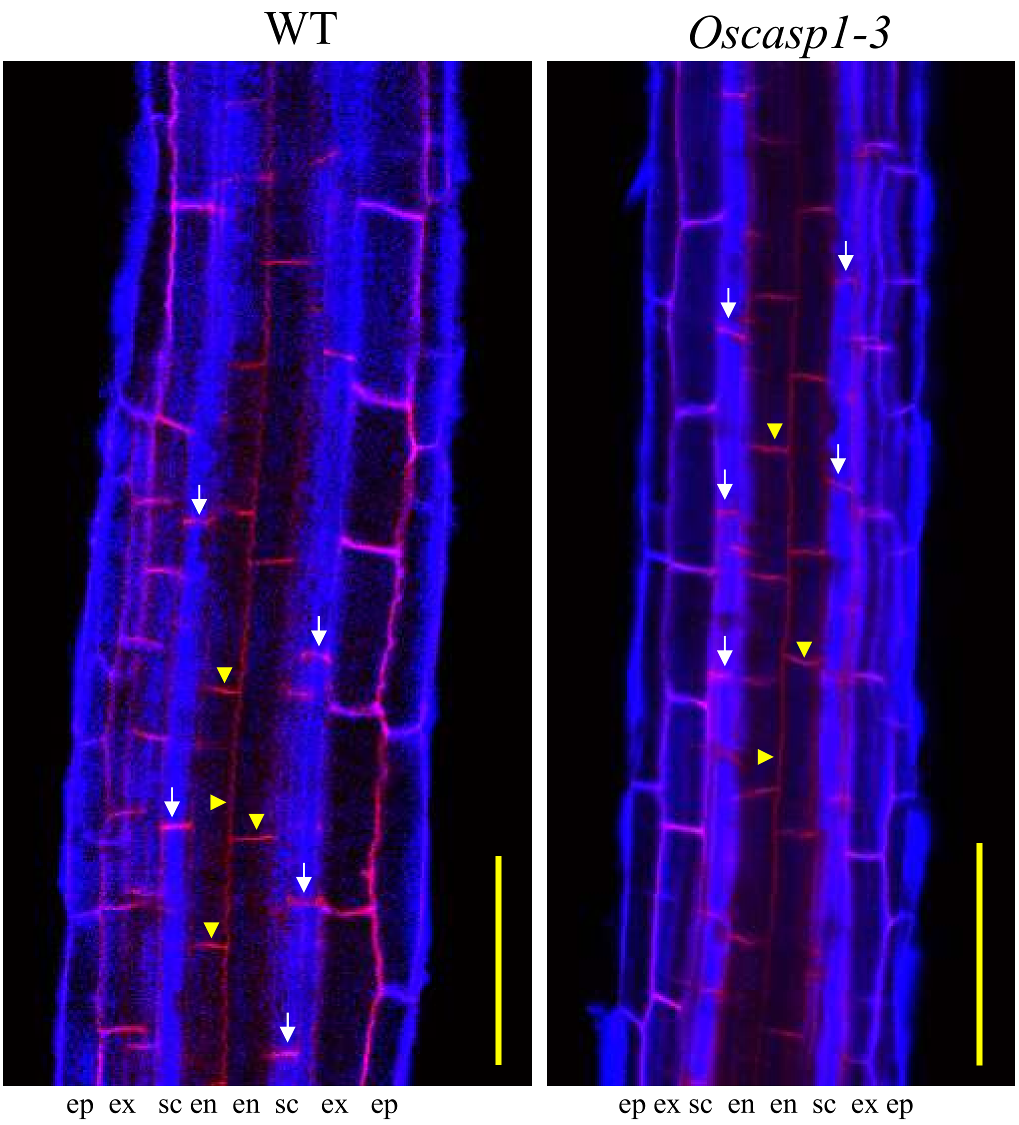

Figure S10. The representative lignin bands in SLRs stained with Basic Fuchsin and Calcofluor White in ClearSee solution. Yellow arrow heads indicate CS bands in the endodermis, and white arrows indicate the lignin bands in the sclerenchyma. ep, epidermis; ex, exodermis; sc, sclerenchyma; en, endodermis. Scale bar = 25μm

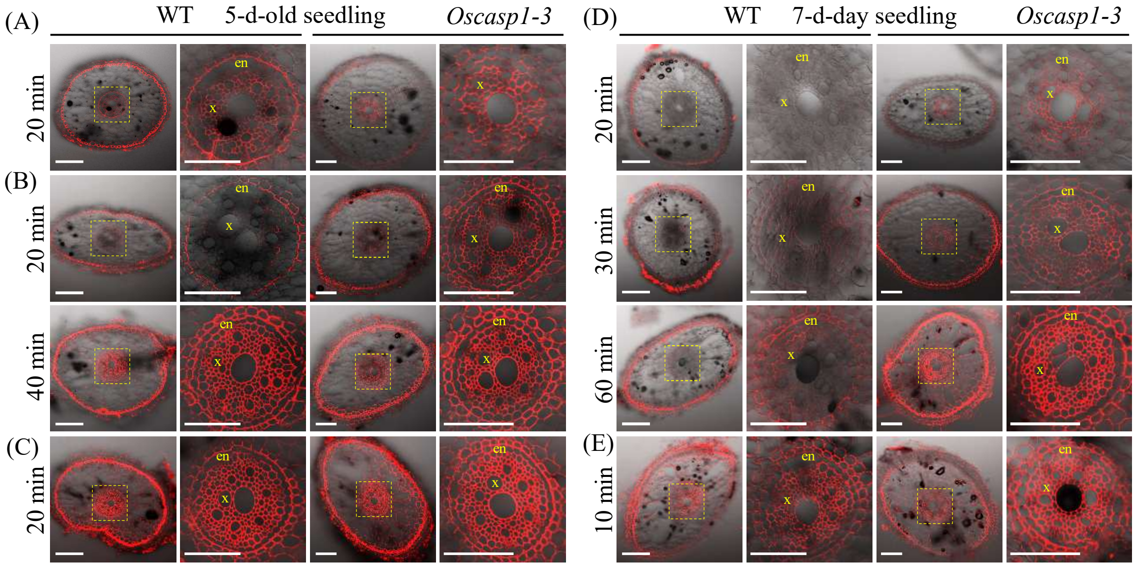

Figure S11. The PI permeability of the *Oscasp1-3* and WT primary roots. Roots of 5-d-old seedlings (A, B, and C) and 7-d-old seedlings (D and E) were incubated in 10 µg/mL PI solution (A, B, and D) or 100 µg/mL (C and E) for the indicated times. Cross-sections of the *Oscasp1-3* mutant and WT primary roots were observed. (A) Cross-sections at 20mm from the root tip. (B, C, D, and E) Cross-sections at 30mm from the root tip. The yellow boxed areas were magnified on the right, en, endodermis, x, xylem, Scale bars = 50 µm.

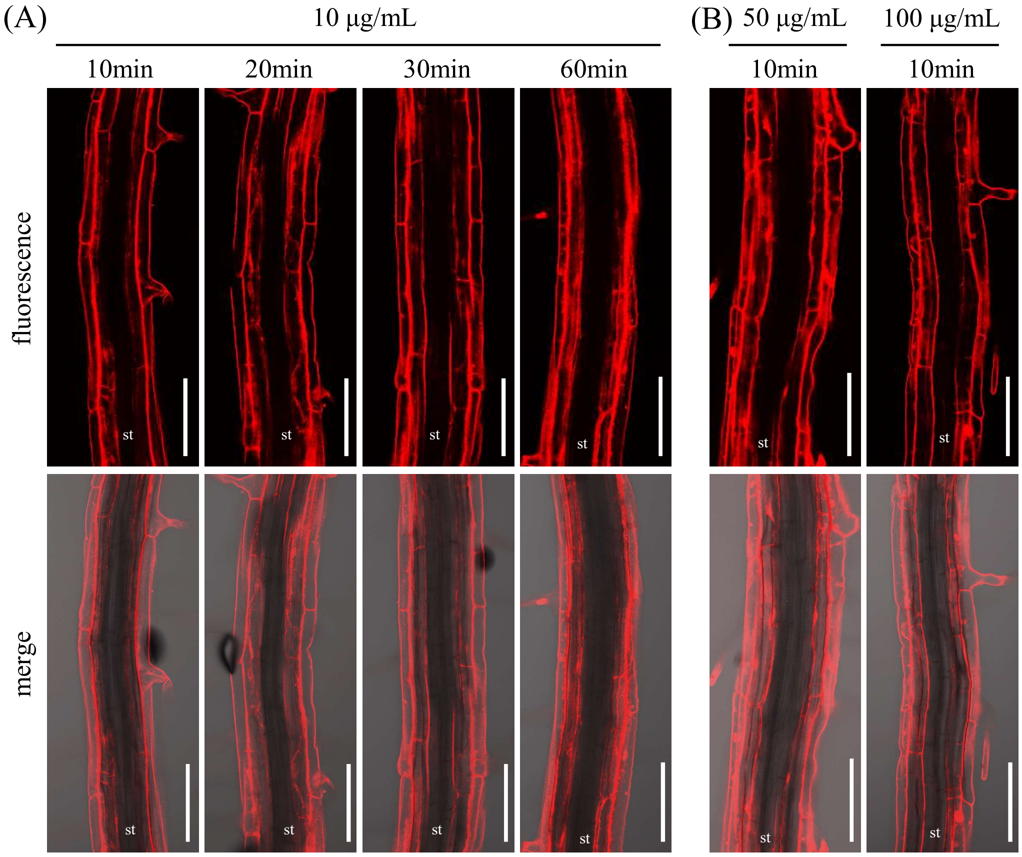

Figure S12. The representative *Arabidopsis* roots (Col-0) incubated with 10 μg/mL PI for different staining times (A) or with higher concentrations for 10 mins (B). st: stele.

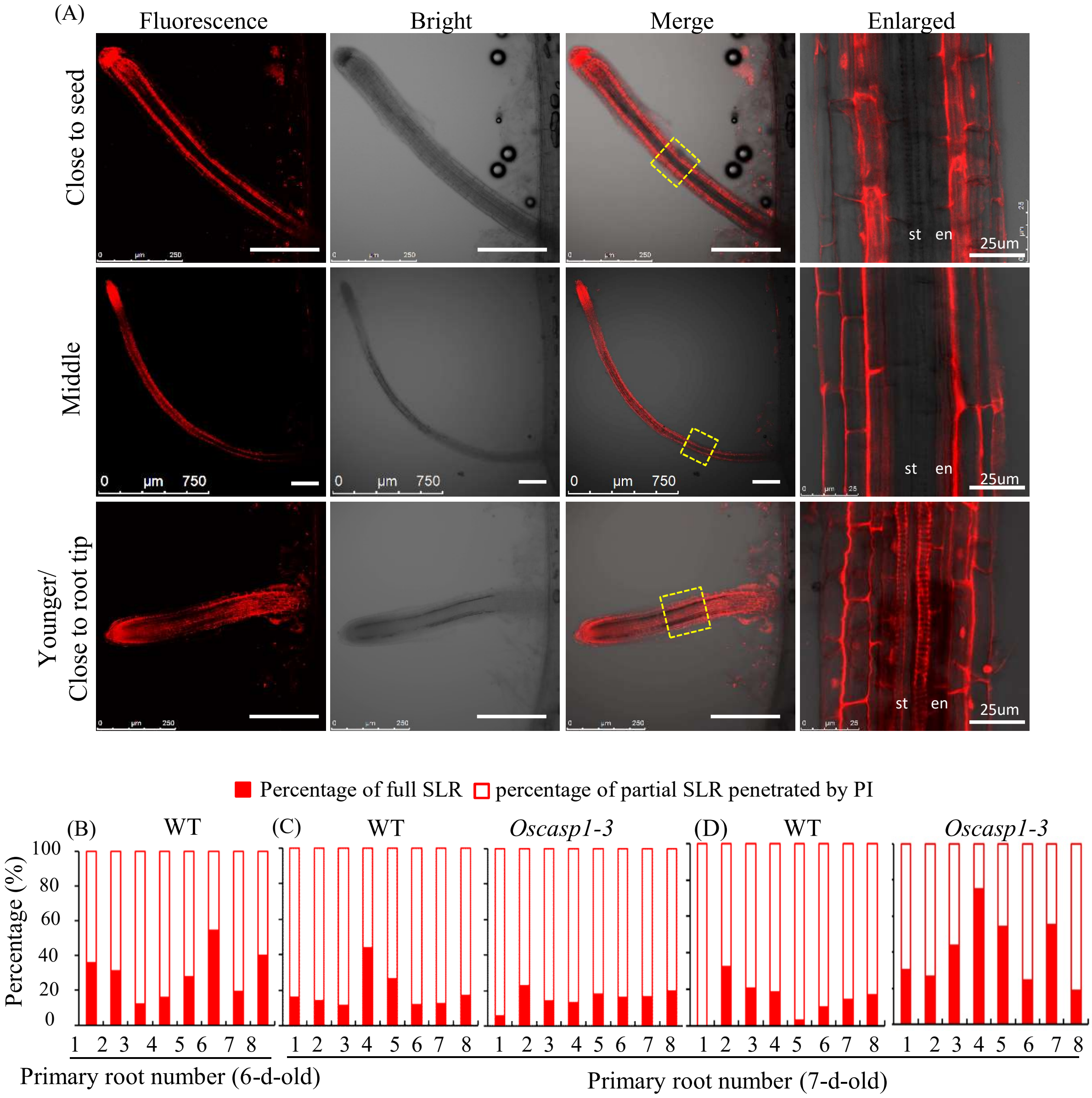

Figure S13. The PI permeability of different SLRs. (A) Representative SLRs located at different positions in WT primary root after staining with PI (10 µg/mL). Scale bar: 200µm and 25µm (enlarged). (B) The percentages of full SLR and partial SLR penetrated by PI in different WT primary roots (6-day-old). Staining time: 10 mins. PI concentration: 10 µg/mL. (C) (D) The percentages of full SLR and partial SLR penetrated by PI in different primary roots (7-days-old). PI concentration: 10 µg/mL. Staining time: 10 mins (C), 60 mins (D).

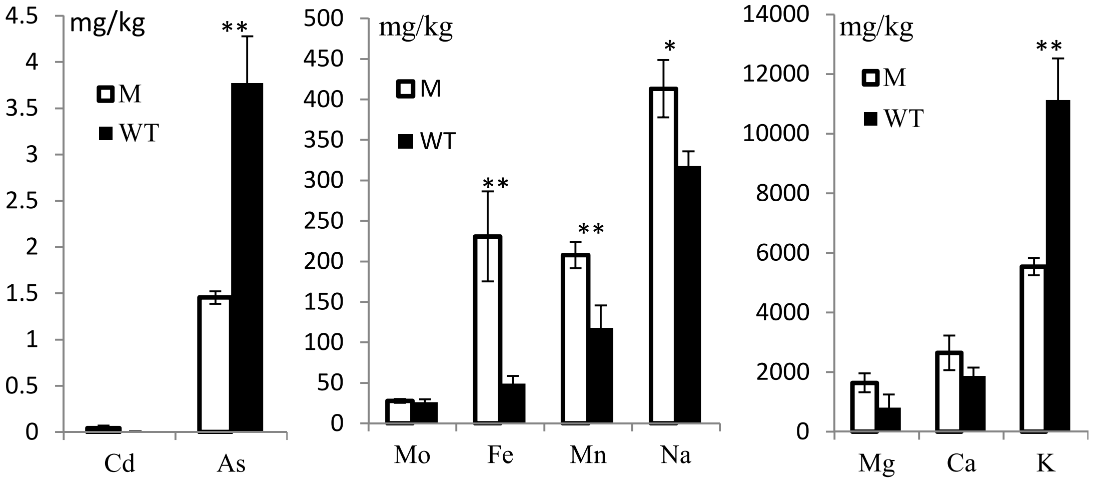

Figure S14. Mineral elemental content in the leaves of the WT and *Oscasp1-3* mutant (g/kg Dry weight). M: *Oscasp1-3*; * and ** indicate the significant differences between the WT and *Oscasp1-3* mutant at 5% and 1% level, respectively.

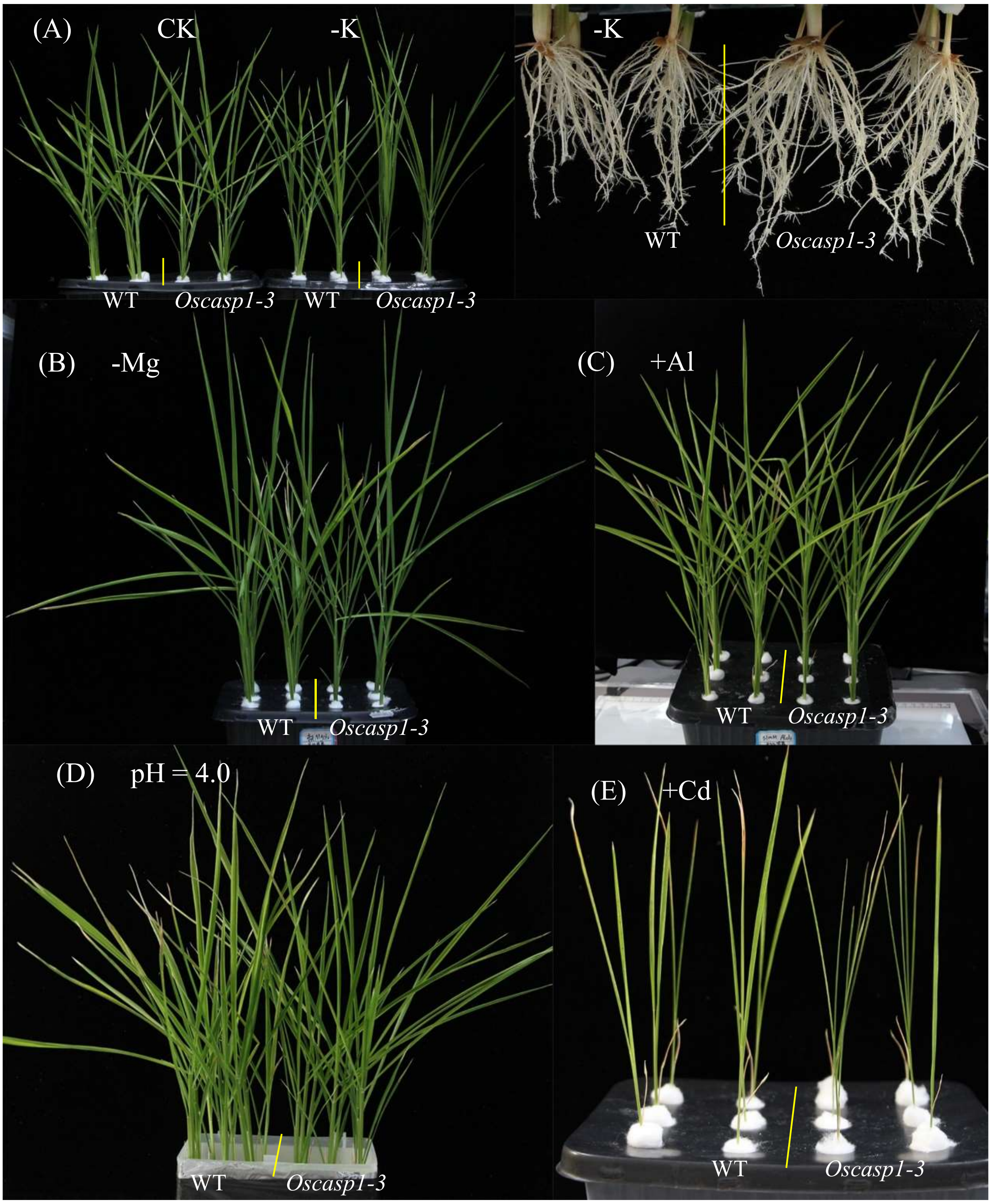

Figure S15. Phenotypes of WT and *Oscasp1-3* mutant plants grown in various media with nutrient imbalances. (A) In complete medium (CK) and without potassium (-K). (B) In medium without magnesium (-Mg). (C) Treatment with 100uM $AlCl_3$. (D) In medium with low pH value (pH = 4.0). (E) in medium with cadmium (80uM).

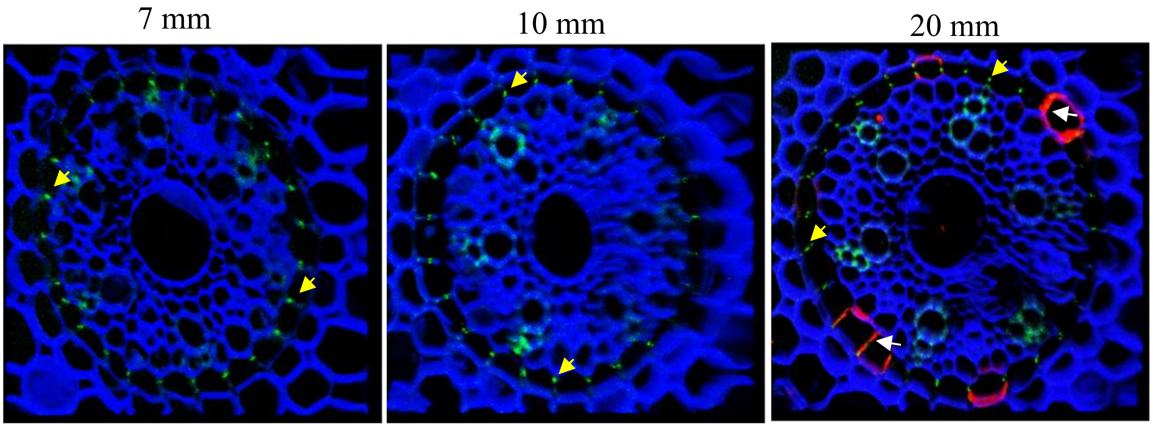

Figure S16. The free-hand cross-sections at 7 mm, 10 mm and 20 mm from the primary root tip of 5-d-old Nipponbare (without *GFP* gene) seedling were detected and observed. Doubling staining (secondary antibody labeled with Alexa Fluor 555 (1:2000 dilution) and Calcofluor White) of the sections were performed for GFP (red) and cellulose (blue) according to the method described by Wang et al (2019). Green fluorescence (an excitation of 488 nm) in endodermis could be attributed to the autofluorescence of CSs. Yellow arrows indicate the possible CSs, and white arrows indicate non-specific binding sites of the GFP antibody (an excitation of 562 nm).
