# Supplementary material for: Rice OsCASP1 orchestrates Casparian strip formation and suberin deposition in small lateral roots to maintain nutrient homeostasis
Source: Front Plant Sci. 2022 Dec 19;13:1007300. doi: 10.3389/fpls.2022.1007300 (PMC9807177; doi:10.3389/fpls.2022.1007300)
Supplement: Supplementary file 2 [file Table_1.docx]

Table S1: Genes encoding the proteins for suberin biosynthesis and CS formation in Arabidopsis and their orthologs in rice.

| Biological  process | Related genes in *Arabidopsis* | Description | Ref | Orthologous genes in rice | Gene description in rice | Ref |
| --- | --- | --- | --- | --- | --- | --- |
| For Casparian strip | Myb36 (AT5G57620) | Myb transcription factor 36 | (1) | Os08g0248700  Os02g0786400  Os03g0771100 | Myb transcription factor 36a, 36b, and 36c | (2) |
|  | CASP1 (AT2G36100)  /CASP2 (AT3G11550) /CASP3 (AT2G27370)  /CASP4 (AT5G06200) | Casparian strip membrane domain proteins | (3) | Os04g0684300  /Os08g0101900 | Casparian strip membrane domain protein |  |
|  | CASP5 (AT5G15290) | Casparian strip membrane domain protein 5 | (3) | Os06g0231050/ Os02g0743900/  Os04g0460400 | Casparian strip membrane domain protein |  |
|  | Rbohf (AT1g64060) | Respiratory burst oxidase homolog f | (4) | Os05t0528000/  Os01t0734200 | OsRBOH5/OsRbohA/OsNOX2 |  |
|  | Per64 (AT5G42180) | Peroxidase required for Casparian strip lignification | (4) | Os02t0833900 | Prx32 |  |
|  | SGN1 (AT1G61590) | Protein kinase | (5) | Os03t0364400 | BBS1 |  |
|  | SGN3 (AT4G20140) | A putative leucine-rich repeat transmembrane- receptor kinase | (6) | Os07t0498400 | A putative leucine-rich repeat transmembrane- receptor kinase |  |
|  | ESB1 (AT2G28670) | ENHANCED SUBERIN 1, Dirigent-like protein | (7) | Os03g0280750; Os01g0155300 | A dirigent-like protein |  |
|  | LOTR1 (AT5G50150) | LORD OF THE RINGS 1, A putative extracellular protease | (8) | Os06g0474500 | A putative extracellular protease |  |
|  | ABCG29 (AT3G16340) | ATP-binding cassette subfamily G transporter 29 | (9) | Os01g0609300 | ATP-binding cassette subfamily G transporter |  |
|  | CIF1 (AT2G16385) | CIF1 is a peptide hormone expressed in the root stele | (10) | Os11g0323860 | CASPARIAN STRIP INTEGRITY FACTOR 1 |  |
|  | CIF2 (AT4G34600) | CIF2 is a peptide hormone expressed in the root stele | (10) | Os11g0323860 | CASPARIAN STRIP INTEGRITY FACTOR 1 |  |
|  |  |  |  |  |  |  |
| **Suberin biosynthesis** | KCS2 (AT1G04220) | β-ketoacyl-CoA synthase 2 | (11) | Os05g0574600  (OsFAE1) | Similar to 3-ketoacyl-CoA synthase. |  |
|  | KCS20 (AT5G43760) | β-ketoacyl-CoA synthase 20 | (11) | Os06g0598800  (WSL1) | β-ketoacyl CoA synthase, Biosynthesis of cuticular waxes on rice leaf |  |
|  | KCS6 (AT1G68530) | β-ketoacyl-CoA synthase 6  (Ortholog of StKCS6 (ACF17125)) | (12) | Os03g0220100 (KCS6/WSL4/CER6) | Very-long-chain fatty acid (VLCFA) elongation | (13) |
|  | FAR1 (AT5G22500) | Fatty acyl-CoA reductase | (14) | Os08g0298700  / Os09g0567500 | A putative fatty acyl CoA reductase |  |
|  | FAR4 (AT3G44540) | Fatty acyl-CoA reductase | (14) | Os08g0298700  / Os09g0567500 | A putative fatty acyl CoA reductase |  |
|  | FAR5 (AT3G44550) | Fatty acyl-CoA reductase | (14) | Os08g0298700 | A putative fatty acyl CoA reductase |  |
|  | CYP86B1/RALPH  (AT5G23190) | Cytochrome P450-dependent fatty acidω-hydroxylase | (15) | Os10g0486100  (CYP86B1) | Cytochrome P450-like protein |  |
|  | CYP86A1/HORST  (AT5G58860) | Cytochrome P450-dependent fatty acidω-hydroxylase | (16) | Os01g0854800  (CYP86A7-2) | Similar to Cytochrome P450 86A1, P450-dependent fatty acid omega-hydroxylase |  |
|  | GPAT5(AT3G11430) | Glycerol-3-phosphate acyltransferase | (17) | Os05g0457800 (OsGPAT) | Similar to Glycerol-3-phosphate acyltransferase 5 |  |
|  | ASFT(AT5G41040) | Feruloyl transferase/ω-hydroxy acid  hydroxycinnamoyl transferase | (18) | Os11g0507200 | Similar to transferase. |  |
| **Secretion** | ABCG1(AT2G39350) | ABCG Transporters are required for Suberin | (19) | Os03g0281900  (OsABCG5/RCN1) | ATP-binding cassette (ABC) transporter, Hypodermal suberization of roots, Salt stress tolerance | (20) |
|  | ABCG2(AT2G37360) | ATP-binding cassette subfamily G transporter 2 | (19) | Os03g0281900  (OsABCG5/RCN1) | ATP-binding cassette (ABC) transporter, Hypodermal suberization of roots, Salt stress tolerance | (20) |
|  | ABCG6(AT5G13580) | ATP-binding cassette subfamily G transporter 6 | (19) | Os03g0281900  (OsABCG5/RCN1) | ATP-binding cassette (ABC) transporter, Hypodermal suberization of roots, Salt stress tolerance | (20) |
|  | ABCG20(AT4G28110) | ATP-binding cassette subfamily G transporter 20 | (19) | Os03g0281900  (OsABCG5/RCN1) | ATP-binding cassette (ABC) transporter, Hypodermal suberization of roots, Salt stress tolerance | (20) |
| **Regulation** | Myb39(AT4G17785) | Myb transcription factor 39 | (21) | Os06g0112700  (OsDLN156) | Similar to Typical P-type R2R3 Myb protein, DLN motif protein 156 |  |
|  | Myb41(AT4G28110) | Myb transcription factor 41 | (22) (23) | Os07g0558100 | Similar to Myb-related transcription factor LBM1, transcription factor MYB7 |  |
|  | Myb53 (AT5G65230) | Myb transcription factor 53 | (23) | Os06g0221000/  Os08g0486300 | Similar to P-type R2R3 Myb protein |  |
|  | Myb92 (AT5G10280) | Myb transcription factor 92 | (23) | Os06g0221000/  Os08g0486300 | Similar to P-type R2R3 Myb protein |  |
|  | Myb93 (AT1G34670) | Myb transcription factor 93 | (23) | Os06g0221000/  Os08g0486300 | Similar to P-type R2R3 Myb protein |  |

Reference

1. T. Kamiya *et al.*, The MYB36 transcription factor orchestrates Casparian strip formation. *Proceedings of the National Academy of Sciences of the United States of America* **112**, 10533-10538 (2015).

2. Z. Wang *et al.*, Three OsMYB36 members redundantly regulate Casparian strip formation at the root endodermis. *The Plant cell* 10.1093/plcell/koac140 (2022).

3. D. Roppolo *et al.*, A novel protein family mediates Casparian strip formation in the endodermis. *Nature* **473**, 380-383 (2011).

4. Y. Lee, M. C. Rubio, J. Alassimone, N. Geldner, A mechanism for localized lignin deposition in the endodermis. *Cell* **153**, 402-412 (2013).

5. J. Alassimone *et al.*, Polarly localized kinase SGN1 is required for Casparian strip integrity and positioning. *Nature plants* **2**, 16113 (2016).

6. A. Pfister *et al.*, A receptor-like kinase mutant with absent endodermal diffusion barrier displays selective nutrient homeostasis defects. *eLife* **3**, e03115 (2014).

7. P. S. Hosmani *et al.*, Dirigent domain-containing protein is part of the machinery required for formation of the lignin-based Casparian strip in the root. *Proceedings of the National Academy of Sciences of the United States of America* **110**, 14498-14503 (2013).

8. B. Li *et al.*, Role of LOTR1 in Nutrient Transport through Organization of Spatial Distribution of Root Endodermal Barriers. *Current biology : CB* **27**, 758-765 (2017).

9. S. Alejandro *et al.*, AtABCG29 is a monolignol transporter involved in lignin biosynthesis. *Current biology : CB* **22**, 1207-1212 (2012).

10. T. Nakayama *et al.*, A peptide hormone required for Casparian strip diffusion barrier formation in Arabidopsis roots. *Science* **355**, 284-286 (2017).

11. S. B. Lee *et al.*, Two Arabidopsis 3-ketoacyl CoA synthase genes, KCS20 and KCS2/DAISY, are functionally redundant in cuticular wax and root suberin biosynthesis, but differentially controlled by osmotic stress. *The Plant journal : for cell and molecular biology* **60**, 462-475 (2009).

12. O. Serra *et al.*, Silencing of StKCS6 in potato periderm leads to reduced chain lengths of suberin and wax compounds and increased peridermal transpiration. *Journal of experimental botany* **60**, 697-707 (2009).

13. X. Wang *et al.*, A beta-Ketoacyl-CoA Synthase Is Involved in Rice Leaf Cuticular Wax Synthesis and Requires a CER2-LIKE Protein as a Cofactor. *Plant Physiol* **173**, 944-955 (2017).

14. F. Domergue *et al.*, Three Arabidopsis fatty acyl-coenzyme A reductases, FAR1, FAR4, and FAR5, generate primary fatty alcohols associated with suberin deposition. *Plant physiology* **153**, 1539-1554 (2010).

15. V. Compagnon *et al.*, CYP86B1 Is Required for Very Long Chain omega-Hydroxyacid and alpha,omega-Dicarboxylic Acid Synthesis in Root and Seed Suberin Polyester. *Plant physiology* **150**, 1831-1843 (2009).

16. R. Hofer *et al.*, The Arabidopsis cytochrome P450 CYP86A1 encodes a fatty acid omega-hydroxylase involved in suberin monomer biosynthesis. *Journal of experimental botany* **59**, 2347-2360 (2008).

17. F. Beisson, Y. Li, G. Bonaventure, M. Pollard, J. B. Ohlrogge, The acyltransferase GPAT5 is required for the synthesis of suberin in seed coat and root of Arabidopsis. *The Plant cell* **19**, 351-368 (2007).

18. I. Molina, Y. Li-Beisson, F. Beisson, J. B. Ohlrogge, M. Pollard, Identification of an Arabidopsis feruloyl-coenzyme A transferase required for suberin synthesis. *Plant physiology* **151**, 1317-1328 (2009).

19. V. Yadav *et al.*, ABCG Transporters Are Required for Suberin and Pollen Wall Extracellular Barriers in Arabidopsis. *The Plant cell* **26**, 3569-3588 (2014).

20. K. Shiono *et al.*, RCN1/OsABCG5, an ATP-binding cassette (ABC) transporter, is required for hypodermal suberization of roots in rice (Oryza sativa). *The Plant journal : for cell and molecular biology* **80**, 40-51 (2014).

21. C. Wang *et al.*, Developmental programs interact with abscisic acid to coordinate root suberization in Arabidopsis. *The Plant journal : for cell and molecular biology* **104**, 241-251 (2020).

22. D. K. Kosma *et al.*, AtMYB41 activates ectopic suberin synthesis and assembly in multiple plant species and cell types. *The Plant journal : for cell and molecular biology* **80**, 216-229 (2014).

23. V. Shukla *et al.*, Suberin plasticity to developmental and exogenous cues is regulated by a set of MYB transcription factors. *Proceedings of the National Academy of Sciences of the United States of America* **118** (2021).
