# Supplementary material for: Rice OsCASP1 orchestrates Casparian strip formation and suberin deposition in small lateral roots to maintain nutrient homeostasis
Source: Front Plant Sci. 2022 Dec 19;13:1007300. doi: 10.3389/fpls.2022.1007300 (PMC9807177; doi:10.3389/fpls.2022.1007300)
Supplement: Supplementary file 4 [file Table_3.docx]

Table S3. Formula of hydroponic nutrient solution used in this study (pH = 5.8).

| Elements | Nutrient salt | Dosage (mM) |
| --- | --- | --- |
| N | NH_4_NO_3_ | 1 |
| P | Na_2_HPO_4_^.^12H_2_O | 0.37 |
| K | K_2_SO_4_ | 0.49 |
| Ca | CaCl_2_ | 0.2 |
| Mg | MgCl_2_^.^6H_2_O | 0.4 |
| Si | Na_2_SiO_3_· 9H_2_O | 1.45 |
| B | H_2_BO_3_ | 0.05 |
| Mn | MnCl_2_^.^4H2O | 9.14 x10^-3^ |
| Cu | CuSO_4_ ·5H_2_O | 1.6 x10^-4^ |
| Zn | ZnSO_4_ ·7H_2_O | 7.46 x10^-4^ |
| Mo | NaMoO_4_· 2H_2_O | 3.72x10^-4^ |
| Fe | Fe-EDTA | 0.02 |
